# A single-base deletion in exon 2 of Hd1 delineates monogenic recessive photoperiod insensitivity in aromatic *Joha* rice: a novel allele for seasonal adaptability

**DOI:** 10.1186/s40659-024-00553-7

**Published:** 2024-11-30

**Authors:** Bodeddula Jayasankar Reddy, Shreekant M. Baradkar, Tamma V. S. S. Manogna, Dibosh Bordoloi, Subhash C. Bihani, Nagendra Sarma Barua, Akhil Ranjan Baruah, Bikram Kishore Das, Suvendu Mondal, Debojit Sarma

**Affiliations:** 1 Department of Plant Breeding and Genetics, Assam Agricultural University, Jorhat, Assam 785013 India; 2 Nuclear Agriculture and Biotechnology Division (NA&BTD), Bhabha Atomic Research Centre (BARC), Trombay, Mumbai, 400085 India; 3 Protein Crystallography Section, Bio-Science Group, Bhabha Atomic Research Centre (BARC), Trombay, Mumbai, 400085 India; 4 Homi Bhabha National Institute, Training School Complex, Anushaktinagar, Mumbai, 400094 India; 5 Department of Agricultural Biotechnology, Assam Agricultural University, Jorhat, Assam 785013 India

**Keywords:** *Joha* rice, Allele-specific marker, Bulk segregant analysis, Gene tagging, Heading date 1, *Oryza sativa* L., Photoperiod insensitivity, Point mutation, SSR marker

## Abstract

**Background:**

Assam's aromatic *Joha* rice is a unique rice class famous for its aroma, taste, and nutritional benefits, which fetch high market prices in domestic and international markets. *Joha* landraces are inherently poor yielders due to their strong aroma and predominantly photoperiod sensitivity. Hybridization involving non-aromatic HYVs improves yield with concomitant loss of quality. In this context, mutation breeding, a sustainable approach where genetic mutations are induced to create desirable traits, often provides useful allelic variation in specific morpho-agronomic traits. The present study delves into the genetic characterization of a photoperiod-insensitive mutant. As part of our mutation breeding programme, this mutant was isolated from a gamma ray-induced M_2_ population of a *Joha* rice landrace, *Kon Joha*.

**Results:**

The mutant was unique, and a single recessive gene conditions the induced photoperiod insensitivity. Mutant gene tagging involved 402 SSR and InDel markers, and later polymorphic markers were used for bulk segregant analysis (BSA) in the F2 population of ‘mutant × *Kalijeera* (distant parent)’. BSA revealed an association between the SSR marker RM527 and this mutant trait. This marker is present on chromosome 6 of the rice genome. Using chromosome 6-specific SSR markers in polymorphic screening and BSA revealed another associated marker, RM19725, for the mutant trait. The genomic interval between RM527 and RM19725 harbors a photoperiod-insensitive gene, Hd1, on chromosome 6. Cloning and sequencing of Hd1 genomic fragments from the parents and mutants revealed a single-base deletion in exon 2, leading to a frameshift mutation in the Hd1 protein. This mutation in exon 2 leads to severe structural abnormalities in the CCT domain of the Hd1 protein that is critical for the interaction of the repressing complex with conserved response elements in the florigen gene under long-day conditions, thereby causing photoperiod insensitivity.

**Conclusions:**

The mutant's pleasant aroma and other quality characteristics, comparable to those of the parent cultivar, hold significant promise. They expand its potential use in a structured breeding programme aimed at developing high-value aromatic *Joha* rice. This rice, resilient to winter- and summer-growing environments and with broad seasonal adaptability, could revolutionize the rice market. The practical value of our research is underscored by this exciting possibility.

**Supplementary Information:**

The online version contains supplementary material available at 10.1186/s40659-024-00553-7.

## Background

Flowering involves the transition of the apical meristem from vegetative to reproductive growth. The timing of flowering in rice is called the heading date. It is one of the critical factors considered for its adaptation in different agroecological situations as well as crop seasons across the globe. Rice flowering is generally promoted by short photoperiods; thus, rice is considered a short-day plant. However, it can be grown under long photoperiod conditions where the adaptation of specific genetic backgrounds due to the accumulation of mutations in different essential genes is considered the primary evolutionary force. Flowering is promoted by heading date 3a (Hd3a) and rice flowering locus T (RFT) under both short-day (SD) and long-day (LD) conditions [1, 2]. Hd3a and RFT1 are expressed diurnally in leaves under SDs and LDs. Both Hd3a and RFT1 proteins move to the shoot apical meristem (SAM), where they interact with the basic leucine zipper (bZIP) transcription factor OsFD (*Oryza sativa* flowering delay) to regulate the expression of two rice orthologs (OsMADS14 and OsMADS15) of the Arabidopsis floral meristem identity gene APETALA1, thus provoking the initiation of primary panicle branch primordia [3, 4].

Furthermore, rice FT-INTERACTING PROTEIN1 (OsFTIP1) is required to export RFT1 from companion cells to sieve elements for further movement to the SAM [5]. The ubiquitin-like domain kinase γ4 (OsUbDKγ4) interacts with OsFTIP1 to modulate its degradation in leaves through the 26S proteasome. This dynamic modulation of OsFTIP1 abundance in leaves by the negative regulator OsUbDKγ4 is essential for regulating florigen transport in rice under LD conditions [6].

Hd3a and RFT are generally positively regulated by early heading date 1 (Ehd1) [7]. However, the upstream regulation of Ehd1 is different under both SD and LD conditions. Under SD conditions, heading date 1 (Hd1) promotes Ehd1 and flowering at night. While Hd1 represses Ehd1 during the day under SD conditions [8], the scenario differs under LD conditions. In LDs, the monocot-specific CCT domain-containing protein 'grain number, plant height and heading date7' (Ghd7) represses Ehd1 and thus flowering [8, 9]. This repression is supported by complex formation among Ghd1, DTH8 and Hd1 during the day under LD conditions [8, 10]. Both Hd1 and Ghd7 contain CCT domains. The CCT domain of Hd1 interacts with the B and C subunits of the nuclear factor Y (NF-Y) complex, such as in OsNF-YB11 (also named GHD8) and OsNF-YC7. This trimeric complex binds to a conserved response element (OsCORE2, containing the CCACA motif) in the Hd3a promoter [11, 12].

The vast collection of rice landraces in India is preserved. Short-grained aromatic rice is a small rice subgroup belonging to the Indica group [13], gaining popularity among global consumers. *Joha* rice is a unique, fragrant rice class grown as winter rice in Assam, India. It is trendy and highly valued due to its quality. It possesses a superfine kernel, distinctive aroma, better cooking qualities, and excellent palatability. The aroma of *Joha* rice cultivars is due to the presence of a non-functional betaine aldehyde dehydrogenase 2 (BADH2), which also lowers grain yield [15, 16]. *Joha* rice's low yield, late maturity, and tall stature generally make it a poor competitor to modern high-yielding varieties, occupying approximately 5 per cent of the *Sali* rice area in Assam, with an average yield of 1.0 -1.5 metric tons per ha [14]. However, its aroma and unique cooking quality allowed a global market for *Joha* rice to enter the European market in 2007. *Joha* rice got the Geographical Indications (GI) tag from the Union Ministry of Commerce, India in 2017 (http://ipindiaservices.gov.in). Photoperiod-insensitive aromatic *Joha* rice cultivars would enable the farmers to increase the cropping intensity by growing two crops per year, offering a viable option to expand and sustain its cultivation for family income and health. The mutant can improve *Joha* rice through crossbreeding and act as pre-breeding material. Furthermore, they can help preserve the unique genetic wealth of this rice class for future generations.

*Kon Joha* is a *Joha* rice type that is typically grown in the *Sali* season (July to November) and flowers under SD conditions. This type of rice genotype will not flower normally if grown during LD (February to June) and thus takes a very long period for its maturation in these off-season periods. Gamma-ray mutagenesis was used to broaden the genetic variability of this *Kon Joha* rice variety. Seven fertile photoperiod-insensitive mutants were isolated while growing a subset of the M_2_ population (approx. 10,000 plant population) during the off-season (February to June). The photoperiod-insensitive mutants (PPISMs) flowered during May, while the other parental-type plants flowered in the 3rd week of October. After verifying its true breeding behavior in M_3_, a particular PPIS mutant (JKOJM-250-22-17-120) was used for molecular characterization. The present research will report on the genetics and molecular nature of this mutation that controls photoperiod insensitivity in this *Joha* rice variety.

## Methods

### Induced mutagenesis for photoperiod insensitivity

A mutation breeding program was used to improve the agronomic characteristics of *Joha* rice through 250 Gy gamma-ray treatment. The parent *Kon Joha* is photoperiod sensitive (PS) and thus does not flower under LD conditions. *Kon Joha* is a small-grain aromatic traditional rice genotype preferred by the Assamese population and is used mainly in traditional foods. The PS aromatic *Joha* rice cultivars did not flower long (13 to 14 h.) May–June summer days at Assam. The commercial photoperiod-insensitive (PI) genotypes flower in LD, and the crop matures by June–July. In 2017, 3000 dry uniform seeds of *Kon Joha* maintained by selfing for two generations were exposed to 250 Gy gamma rays from a Cobalt-60 source at Bhabha Atomic Research Centre (BARC), Trombay. The M_1_ generation and the parent were raised as a transplanted crop in two adjacent blocks during early *Ahu* (February to May) 2017. The field emergence rate in the nursery was 37.40% for the treated seeds and 41.13% for the control seeds. However, the crop started flowering on October 17, 2017. All 390 surviving M_1_ plants bearing panicles were grown in the plant to-rows along with intermittent controls during early *Ahu*, 2018. Flowering occurred in nine plants of progeny no. 22 from May 21 to June 7, and seven fertile panicle-bearing plants were confirmed for their flowering behavior and selected for spikelet fertility through the M_3_ to M_5_ generations. The present study used a photoperiod insensitive mutant (PPISM), JKOJM-250-22-17-120, which has the highest spikelet fertility (88.47%).

### Test of genome integrity in isolated mutant compared to available PI rice

Gamma irradiation results in a variety of random DNA damage events, from modest deletions of 1–16 bp to massive deletions of 9.4–129.7 kb and inversions of 1–3208.5 kb [17, 18]. These processes result in various changes, from point mutations to chromosome aberrations, which ultimately influence the morphological, physiological, and anatomical characteristics of the variety or cultivar being improved [19]. Furthermore, Fu et al. [20] suggested that true induced mutants could be differentiated from outcrossing-derived contaminants through microsatellite marker analysis. The SSR banding patterns of true mutants should be identical to those of their immediate parent. Hence, the genome integrity of the isolated mutant in combination with the parent was tested, including two already available photoperiod-insensitive rice cultivars (*Ahu Joha*-1 & *Ahu Joha*-2) and a distant parent, *Kalijeera,* with 150 SSR and InDel [21] markers (Supplementary Table S1). The morphological similarity of the mutants and parents was also tested by observing 54 DUS characteristics and 12 morphometric traits [22, 23].

### Inheritance study of induced photoperiod insensitivity

To assess the pattern of photoperiod insensitivity inheritance in the PPIS mutant, it was hybridized with the parent cultivar (*Kon Joha*) and distant parent, *Kalijeera,* in the winter/*Sali* season of 2019*.* We chose the distant parent to discover more polymorphic markers. F_1_ plants were grown in winter/*Sali*, 2020, to obtain sufficient F_2_ seeds. To expose the plants to two different periods of daylight, i.e., long days (March to September) and short days (October to February), F_2_ generations of both crosses were evaluated in two continuous seasons, referred to as early autumn/*Ahu*, 2021, to winter/*Sali*, 2021. A total of 250 F_2_-derived F_3_ (F_2:3_) progeny rows from the cross PPIS mutant × *Kon Joha* were studied in early autumn, 2022, to further demonstrate the genotypic ratio of photoperiod insensitivity by observing photoperiod-insensitive plants in F_2:3_ progeny rows. The resulting F_2_ and F_2:3_ data were subjected to the χ^2^ test for their goodness of fit with the predicted 3:1 and 1:2:1 Mendelian segregation ratios, respectively. During the confirmation of the genotypic ratio in F_3_, gDNAs from an individual of 10 true bred wild-type and 10 true bred mutant progenies were isolated to validate candidate gene-derived allele-specific SNP markers.

### Bulk segregant analysis (BSA) for the identification of linked SSR markers

From the F_2_ population of ‘PPIS mutant × *Kalijeera*’, 10 photoperiod-insensitive F_2_ plants and 10 photoperiod-sensitive F_2_ plants were chosen for genomic DNA isolation, and photoperiod-insensitive bulk (PIS bulk) and photoperiod-sensitive bulk (PS bulk) plants were subsequently prepared by pooling equal molar concentrations of gDNA from each plant. Initially, 402 SSR and InDel markers (Supplementary Table S1) were screened for polymorphisms between the PPIS mutant and *Kon Joha* and *Kalijeera strains*. Only polymorphic markers were used for bulk segregant analysis [24]. Based on the BSA results, chromosome 6-specific SSR markers were synthesized from the genomic interval of 5.0 to 13.2 Mbp for fine mapping (Supplementary Table S2).

### Whole-genome resequencing of parents and mutants

Total DNA was isolated from *Kon Joha* and PPIS mutants following the standard CTAB method, and the quality and quantity of the samples were ascertained by resolving on a 1.2% agarose gel and with a Nanodrop1000 spectrophotometer (Thermo Fisher Scientific, Waltham, MA, USA), respectively. The DNA samples were then sequenced at 30X coverage on the Illumina HiSeq2500 platform (Nucleome Informatics Pvt. Ltd., Hyderabad, India). After filtering the clean reads, the *Kon Joha* sequences were aligned to the reference genome *Oryza sativa* ssp*. Japonica* cv. Nipponbare (MSU7). The alignments are reported in bam (binary alignment and mapping) format using BWA with the default parameters. InDels were marked if they were ≤ 50 bases in size. The consensus sequence for wild-type '*Kon Joha*’ was generated using SAMtools [25]. Later, the filtered PPIS mutant sequences were mapped against the consensus *Kon Joha* sequence. Genotyping by calling variants was performed to obtain better support and confidence from BCFtools. These variants are emitted in a cumulative form in a VCF file (Variant Calling Format), which is then strictly filtered using BCFtools v1.7, which helps to eliminate false variants created during mapping or variant calling. SNPs were extracted from the identified variants and then annotated using the SnpEff tool [26].

### Cloning of the Hd1 gene and sequencing

The specific primer pairs described in Table 1 amplified the Hd1 gene from parent and mutant genomic DNA; the Q5® High-Fidelity DNA Polymerase (New England Biolabs, USA) yielded five overlapping fragments. PCR was performed for 35 cycles (30 s at 94 °C, 30 s at 55 °C, and 30 s at 72 °C), followed by a final amplification for 10 min at 72 °C. PCR amplification products were size-separated using a 1.5% agarose gel at 75 V. The Wizard® SV Gel and PCR Clean-Up System (Promega, Madison, USA) was used to purify the PCR products from the gel. The DNA fragment was treated with Taq DNA polymerase in excess dATP to obtain dA overhangs at 3^/^end. The amplified product was then purified using a High Pure PCR Cleanup Micro Kit (Roche, USA) and ligated into the pTZ57R vector (Thermo Scientific, USA) according to the manufacturer's instructions for transforming *Escherichia coli* DH5α cells. Transformed colonies containing the cloned fragment were identified following standard blue‒white screening and colony PCR. Plasmid DNA was isolated from overnight cultures of positive colonies using the GenElute™ Plasmid Miniprep Kit (Sigma‒Aldrich, USA). The insertion of the recombinant plasmid was confirmed through double digestion with HindIII and EcoRI. Plasmid DNA samples were then subjected to Sanger DNA sequencing. The complete assembled sequence of the Hd1 gene from the parent and mutant Hd1 genes was aligned using CLUSTAL W [27].

**Table 1:** Primer pairs used for cloning and detection of mutations in Hd1

Primer name	Sequence (5′-3′)	Annealing temp. (^o^C)	Product Size (bp)
Primer pairs for cloning of Hd1 genomic fragment			
Hd1_FP1	AAAGCAAAGATGAACAGAGGTG	57.0	700
Hd1_RP1	AGAGATTTTACAGGGATCAATAG		
Hd1_FP2	GAGATGTCCATTGAATTGTTTAG	60.4	701
Hd1_RP2	CCTTGCTTGTGGTAGTAGTAG		
Hd1_FP3	GAGGACAAACACAATAGCTTG	57.0	938
Hd1_RP3	AGCTAGTAATAGATGAACTCAC		
Hd1_FP4	ATAGTGGTTATGGAGTTGTG	62.0	944
Hd1_RP4	GTTTCATAACGTATTGTCTTCTC		
Hd1_FP5	CTGGAGCAATCAATCTCTTC	57.0	1095
Hd1_RP5	CTCATGGTTTAGTGAGAAGATAG		
Allele-specific primer pairs for the nonsynonymous frameshift mutation in Hd1			
Hd1_Wild_FP	AGGGAGGCCAGGGTGCTCATG	66.5	200
Hd1_common RP	CTACTGTCAGATAGAGCTGCAGTGGAGAACATC		
Hd1_Mutant_FP	GGGAGGCCAGGGTGCTCCGT	66.0	200
Hd1_common RP	CTACTGTCAGATAGAGCTGCAGTGGAGAACATC		

### Development of allele-specific SNP markers and marker validation

Based on the sequencing of Hd1, a single base deletion was found in exon 2. Allele-specific SNP markers were developed through WebSNAPPER tools (Supplementary Table S3) [28]. The first pair of oligonucleotides could differentiate both the parent and the mutant for the wild-type allele-specific primer. We synthesized two sets of forward primers to identify mutant allele-specific primers; the second oligonucleotide clearly differentiated the wild type from the mutant (Supplementary Table S3; Table 1). After confirming the polymorphic reaction in the wild type (both *Kalijeera* & *Kon Joha*) and mutant, the allele-specific markers were used for marker validation in the component wild type and mutant type bulk of the ‘mutant × *Kalijeera*’ F_2_ population. Similarly, SNP-based PCR markers were also used for confirmation in 10 homozygous wild-type plants and 10 homozygous mutant-type plants of the ‘mutant × *Kon Joha*’ F_3_ population.

### Structural analysis of wild-type and mutant Hd1 proteins

Three-dimensional structures of wild-type and mutant Hd1 proteins were predicted using AlphaFold2 [29]. AlphaFold2, as implemented in ColabFold, was used for the computational modeling [30]. The modeled structure of only the CCT domain was compared to the crystal structure of the CCT domain (PDB ID 7C9O). Structural analysis and illustrations were generated using the PyMOL molecular graphics system (v.2.6.0a0; Schrodinger, LLC), an open-source molecular visualization software. The sequence alignment figure was prepared using the ESPript server (https://espript.ibcp.fr; [31]).

## Results

### Inheritance of photoperiod insensitivity

**Fig. 1:**
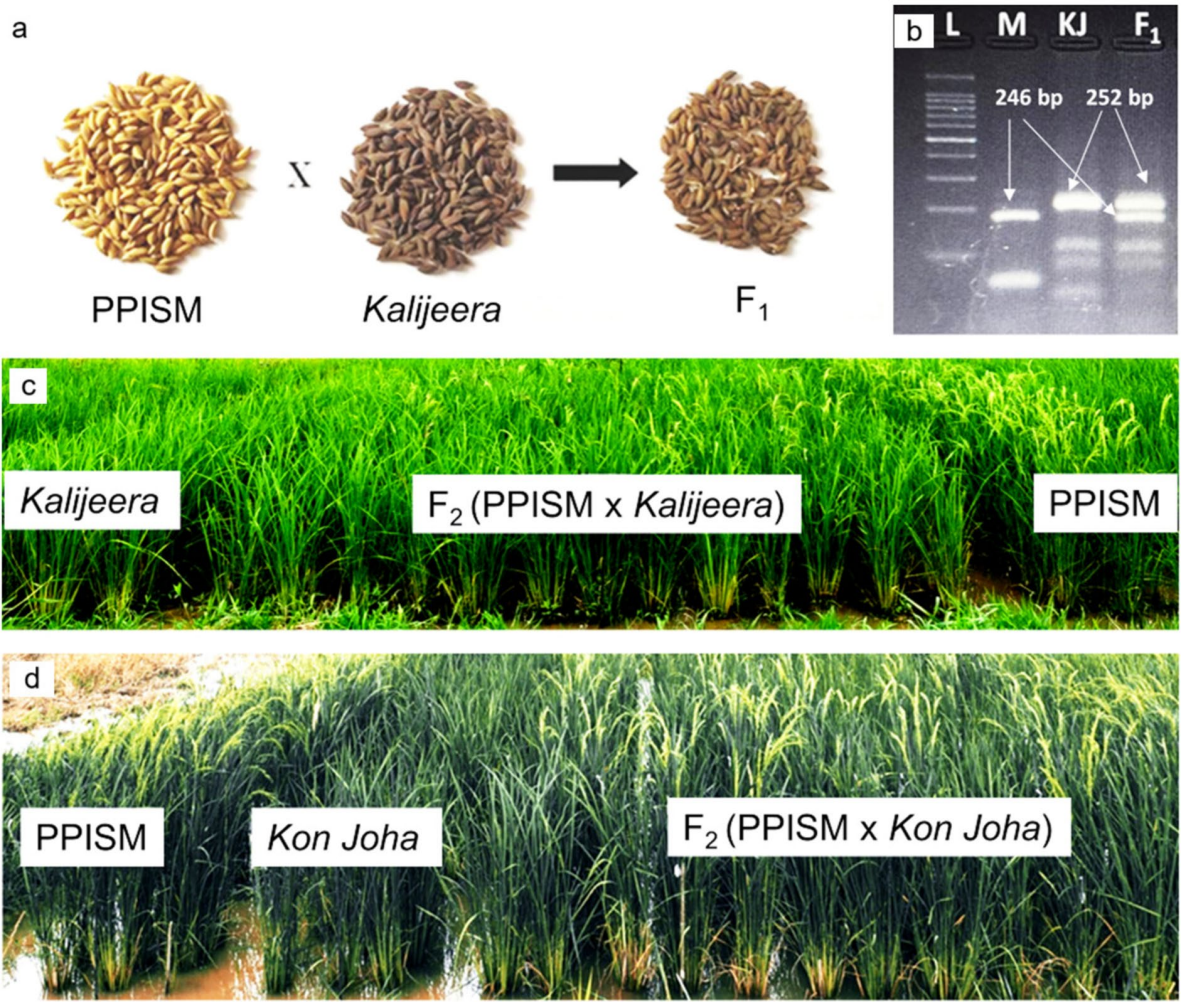
Hybridity confirmation in F_1_ individuals of the cross PPISM/*Kalijeera*: **a** intermediate husk color of F_2_ seeds; **b** RM480 amplified both the bands of mutant (M) and *Kalijeera (KJ)* in F_1_; **c** sensitive *Kalijeera* (left), PPISM (right) and segregating F_2_ of their cross (center); **d** sensitive *Kon Joha* (left), PPISM (right) and segregating F_2_ of their cross (center)

Inheritance of the mutant trait in the PPIS mutant was confirmed in both the F_2_ (Fig. 1) and F_3_ generations of the hybrids ‘PPIS mutant × *Kon Joha*’ and ‘PPIS mutant × *Kalijeera*’. Of the 514 plants in the F_2_ population of ‘PPIS mutant × *Kon Joha*’, 381 were photoperiod sensitive, and 133 were photoperiod insensitive (χ^2^ = 0.21; P = 0.647). Similarly, among the 502 F_2_ plants of the ‘PPIS mutant × *Kalijeera*’, 364 and 138 plants were photoperiod sensitive and photoperiod insensitive, respectively (χ^2^ = 1.66; P = 0.197). When 250 F_2_ progenies (66 photoperiod-insensitive: 184 photoperiod-sensitive) of ‘PPIS mutant × *Kon Joha*' were tested in the F_3_ generation, all 66 photoperiod-insensitive mutant progenies were found to be true bred. A total of 122 F_2_ plant progenies were heterozygous (3:1), and the remaining 62 plants were subjected to true breeding for photoperiod sensitivity [χ^2^ (1:2:1) = 0.272; P = 0.873]. Thus, F_2_ and F_3_ segregation confirmed the single recessive gene inheritance pattern for this photoperiod insensitivity trait in the PPIS mutant. Plotting the graphs using data on days to flowering of parents and F_2_ individuals of both the PPIS mutant/*Kon Joha* and PPIS mutant/*Kalijeera crosses* resulted in a bimodal distribution curve (Supplementary Figs. 1 & 2). Two peaks were observed for the flowering of F_2_ individuals in response to photoperiod: one for the photoperiod-insensitive group and the other for the photoperiod-sensitive group (Supplementary Figs. 1 & 2). These results clearly indicated a monogenic inheritance pattern for photoperiod insensitivity.

### Genome integrity analysis and genome sequencing revealed more point mutations than repeat length/indel variation

**Fig. 2:**
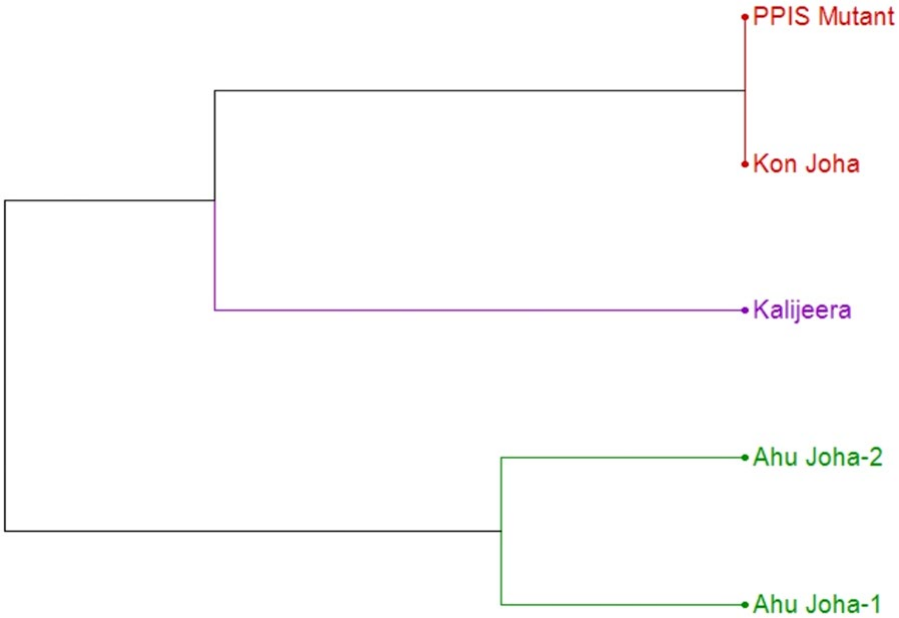
Dendrogram depicting the genetic relationship of the PPIS mutant with the parent and other *Ahu Joha* genotypes

Based on genome integrity analysis of the mutant, parent (*Kon Joha*), *Kalijeera* and photoperiod-insensitive *Ahu Jiha* rice genotypes with 150 SSR and InDel markers (Supplementary Table S1), none of the markers could detect any polymorphisms between the parent and PPIS mutant. In contrast, the PPIS mutant was distinct from the already available photoperiod-insensitive *Ahu Joha* rice cultivars; this established the novelty of the isolated mutant (Fig. 2**)**. Fifty-four descriptors comprising traits at the seedling, vegetative, flowering and maturity phases were recorded (Supplementary Table S4) for the PPIS mutant and *Kon Joha*. The PPIS mutant was monomorphic and similar to *Kon Joha* in terms of fifty-two traits. Polymorphism was evident for the other two traits: the time of heading and maturity (Supplementary Table S4). However, the mutant was polymorphic to *Kon Joha* for filled grain panicle^−1^, plant height (cm) and panicle length (cm) (Supplementary Table S5). These morphological differences may arise due to variations in the environment during flowering in the mutant and parent plants.

Genome sequencing of the parent and mutant revealed as many as 3,57,562 nucleotide variations between the *Kon Joha* and PPIS mutants. Genome sequencing generated 161,333,716 reads (29.5% duplicated data) in *Kon Joha* and 165,632,496 reads (33.7% repeated data) in the PPIS mutant. The overall GC content was 44.4%. Of the 3,57,562 nucleotide variants, 23.17%, 72.42% and 4.41% were silent, missense and nonsense mutations, respectively. Among these missense and nonsense mutations, 8.68% were in the exon region. The average mutation density was 1 in 1046 bases. The Ts/Tv ratio for all the point mutations was 2.38.

### BSA identified two closely spaced SSRs on chromosome 6 linked to the mutant trait

**Fig. 3:**
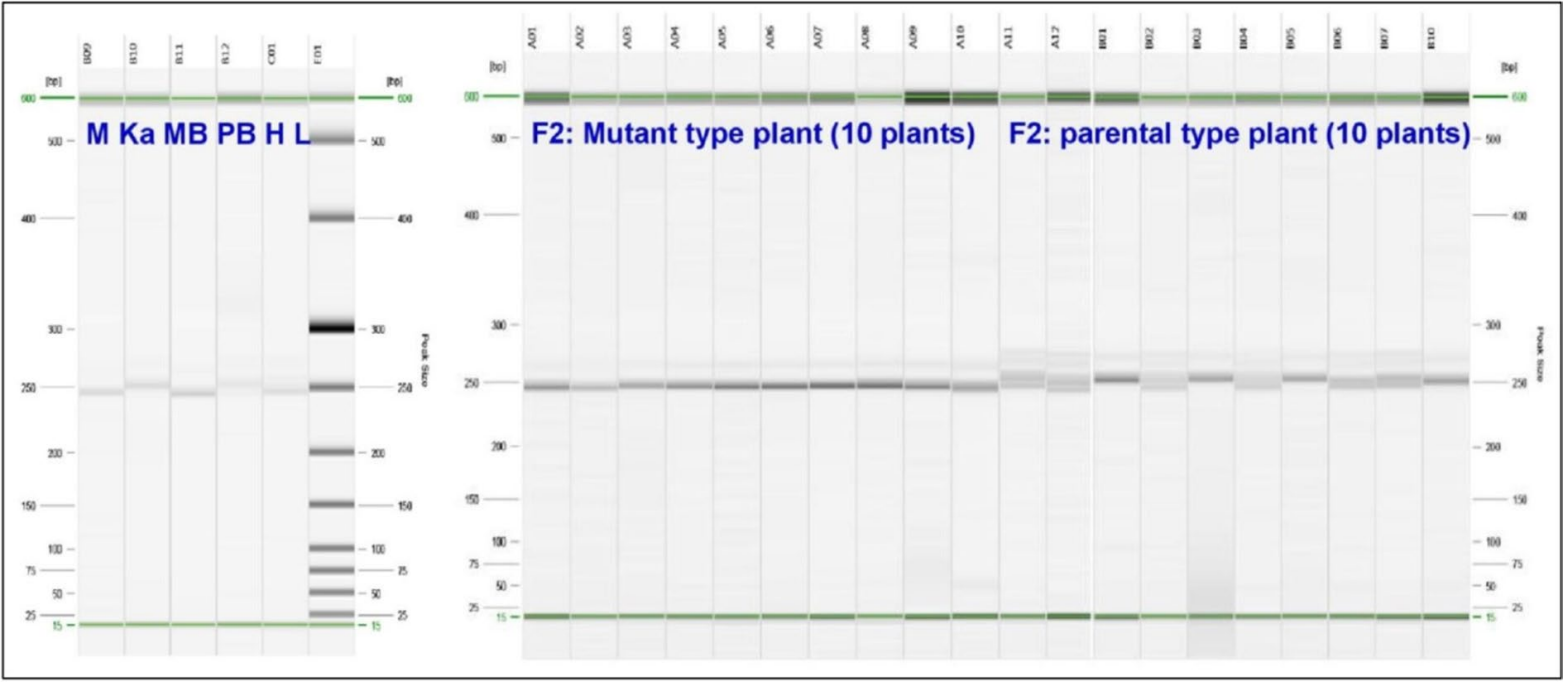
Capillary gel electrophoresis of the RM527 marker in the BSA experiment and bulk F2 individuals of ‘Mutant × *Kalijeera*’

**Fig. 4:**
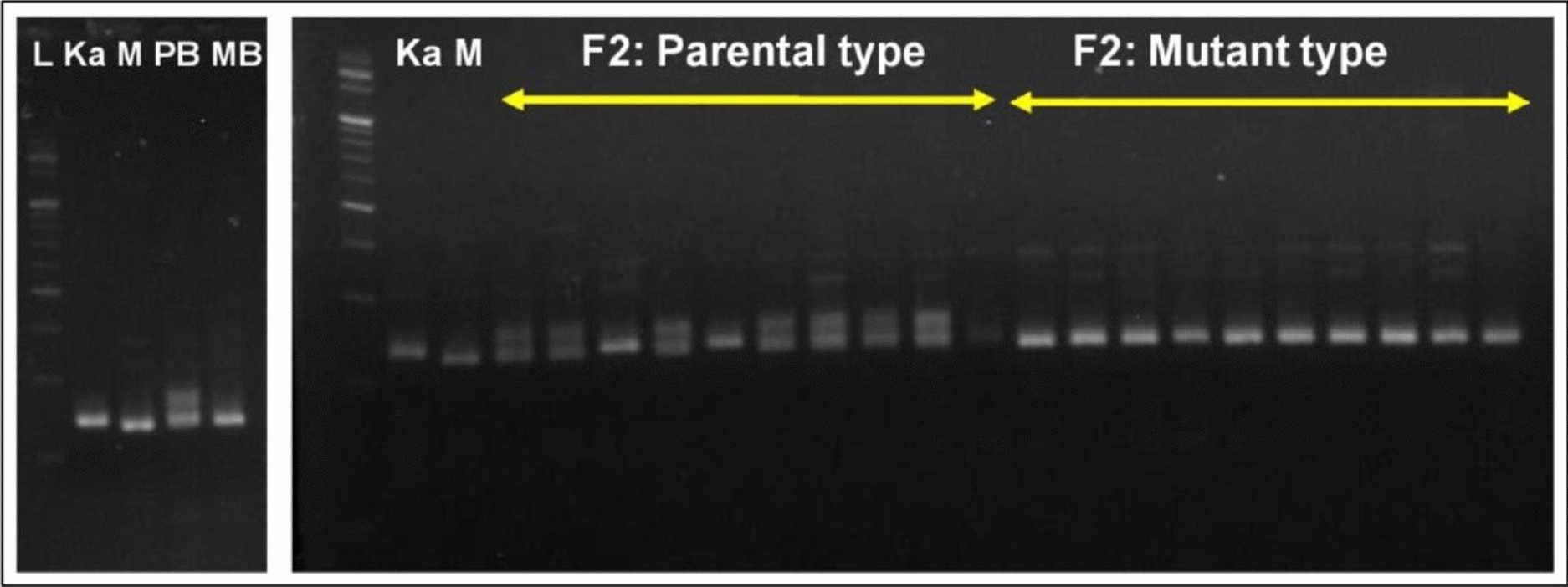
Agarose gel electrophoresis of the RM19725 marker in the BSA experiment and bulk individuals of F_2_ of ‘Mutant × *Kalijeera*’

For quick mapping of the mutant trait in the PPIS mutant, it was hybridized with a distant parent (*Kalijeera*) to develop a segregating F_2_ population. Screening of 402 SSR and InDel markers revealed 57 (15.17%) polymorphic markers between the mutant and *Kalijeera strains*. The BSA of these polymorphic markers in the photoperiod-sensitive bulk (PS bulk) and photoperiod-insensitive bulk (PIS bulk) samples revealed a close association between RM527 and the mutant trait on chromosome 6. Moreover, other SSR markers (RM204, RM225, RM586, RM587, RM588, and RM589) on chromosome 6 were not associated with the mutant trait. The RM527 marker lies between 9,862,291 and 9,862,523 bp on chromosome 6 of rice. Other markers remained at the proximal end of chromosome 6 (beyond 5.0 Mbp). We then synthesized 40 additional SSR markers between 5.0 and 13.2 Mbp on chromosome 6 (Supplementary Table S2). By screening for polymorphisms between *Kalijeera* and the PPIS mutant, five additional SSR markers (RM3794, RM19592, RM19725, RM5850 and RM19902) were found to be polymorphic (Supplementary Table S2). Furthermore, BSA revealed a close association between RM19725 (8,134,068 to 8,134,111 bp in chromosome 6) and the mutant trait. Thus, BSA delineated a marker interval (RM19725—RM527) on chromosome 6 that is tightly associated with photoperiod insensitivity in the PPIS mutant (Figs. 3 & 4). To confirm this, we genotyped all 10 individual mutant-type plants (constituting the PIS bulk) with polymorphic SSR markers (RM589, RM225, RM3794, RM19592, RM19725, RM527, RM5850 and RM19902) on chromosome 6. The genotyping assay in these individual 10 PIS plants revealed no crossing over for RM527 and RM19725, whereas all the other polymorphic SSR markers on chromosome 6 showed recombination and proved their incomplete linkage with a photoperiod-insensitive gene in the PPIS mutant (Supplementary Fig. 3).

### A single-base deletion in Hd1 between RM19725 and RM527

**Fig. 5:**
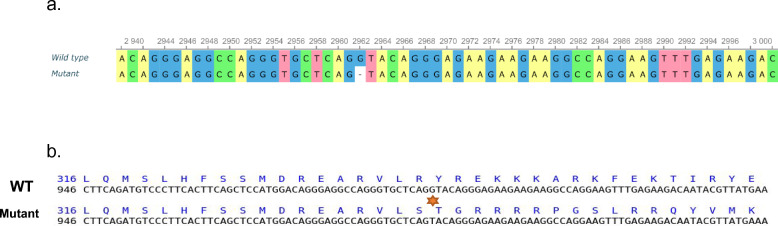
ClustalW sequence alignment of parental and mutant Hd1 surrounding the mutation site. **a** It represents the genomic sequences, where at 2962 position, a single base deletion (G to −) occurred. **b** It represents corresponding triplets match of the ORF

Several flowering-related genes were located on rice chromosome 6. The Hd17 (OsELF3), Hd3a, and RFT1 genes are between 2.23 Mbp and 3.0 Mbp long. Another three genes, Os06g40080/Se5, Os06g41090/OsFTIP and Os06g45640/OsNF-YC4*, were* present at 23.85 Mbp to 27.64 Mbp. Heading date 1 (Hd1) included 9,336,359 to 9,338,643 bp. Hd1 lies perfectly between the identified markers RM19725 and RM527 on chromosome 6. After cloning, we amplified Hd1 from *Kon Joha* and PPIS mutants and sequenced four gene fragments. After aligning the complete sequences of *Kon Joha* (OR113693) and the PPIS mutant (OR113694), a single-base deletion (G to −) was identified in exon 2 of Hd1 (Fig. 5).

### Validating the relationship between mutant traits and candidate gene mutations in two segregating populations

From the above pattern of mutations in Hd1, we could visualize the nonsynonymous nature of single-base deletions in exon 2. The single-base deletion leads to a frameshift mutation phenomenon and a possible increase in nonfunctional proteins. Using the Web-SNAPPPER tool, two allele-specific forward primers were designed for the parent (wild type) and mutant (PPIS mutant) strains, along with a standard reverse primer. The amplification of both of these allele-specific primer pairs was first confirmed in *Kon Joha* and PPIS mutants (Supplementary Fig. 4 & 5). The wild-type allele-specific primer pairs amplified a 200 bp band in *Kon Joha* but not in the PPIS mutant. Mutant-allele-specific primer pairs amplified the band in mutants but did not amplify the band in *Kon Joha*. For validation, two different populations were used. Initially, individual gDNA of bulk components of the BSA study in F_2_ of ‘PPIS mutant × *Kalijeera* (distant parent)' was used. The bands of all 10-individual mutant-type plants were amplified with mutant-allele-specific primer pairs, but no amplicons were amplified with parental-allele-specific primer pairs (Supplementary Fig. 6 & 7). The bands of seven out of the 10 wild-type plants were amplified with mutant allele-specific primer pairs, and the 200 bp amplicons of all 10 plants were amplified with parental allele-specific primer pairs (Supplementary Fig. 6 & 7). The amplification of the band in seven wild-type plants in the F_2_ population of ‘PPIS mutant × Kalijeera’ was due to their heterozygous nature, as revealed by the amplification of the RM19725 marker (Fig. 4). The heterozygosity shown through the allele-specific SNP marker was well matched with the RM19725 amplification due to the close physical proximity with Hd1. The use of allele-specific SNP primer pairs further confirmed the heterozygosity and homozygosity of wild-type F_2_ plants of ‘mutant × *Kalijeera*’. Of the 10 plants, 7 were heterozygous wild type, and 3 were homozygous. Thus, the segregation ratio in the wild-type pool (10 plants) for heterozygous and homozygous plants maintained genotypic segregation at 2:1 (χ^2^ = 0.13, P value = 0.937). For the next level of validation, we used true breeding (deduced from F_3_ segregation), wild-type (10 plants), and mutant-type (10 plants) plants from ‘PPIS mutant × *Kon Joha*’. Due to the homozygosity of the plants, all the wild-type plants amplified bands with parental-allele-specific primer pairs but no bands with mutant-allele-specific primer pairs (Supplementary Fig. 4). The bands of all 10 mutant-type plants were amplified with mutant allele-specific primer pairs, but no bands were amplified with parental allele-specific primers (Supplementary Fig. 5).

### A frameshift in exon 2 of Hd1 leads to drastic changes in the C-terminal region of the Hd1 protein

**Fig. 6:**
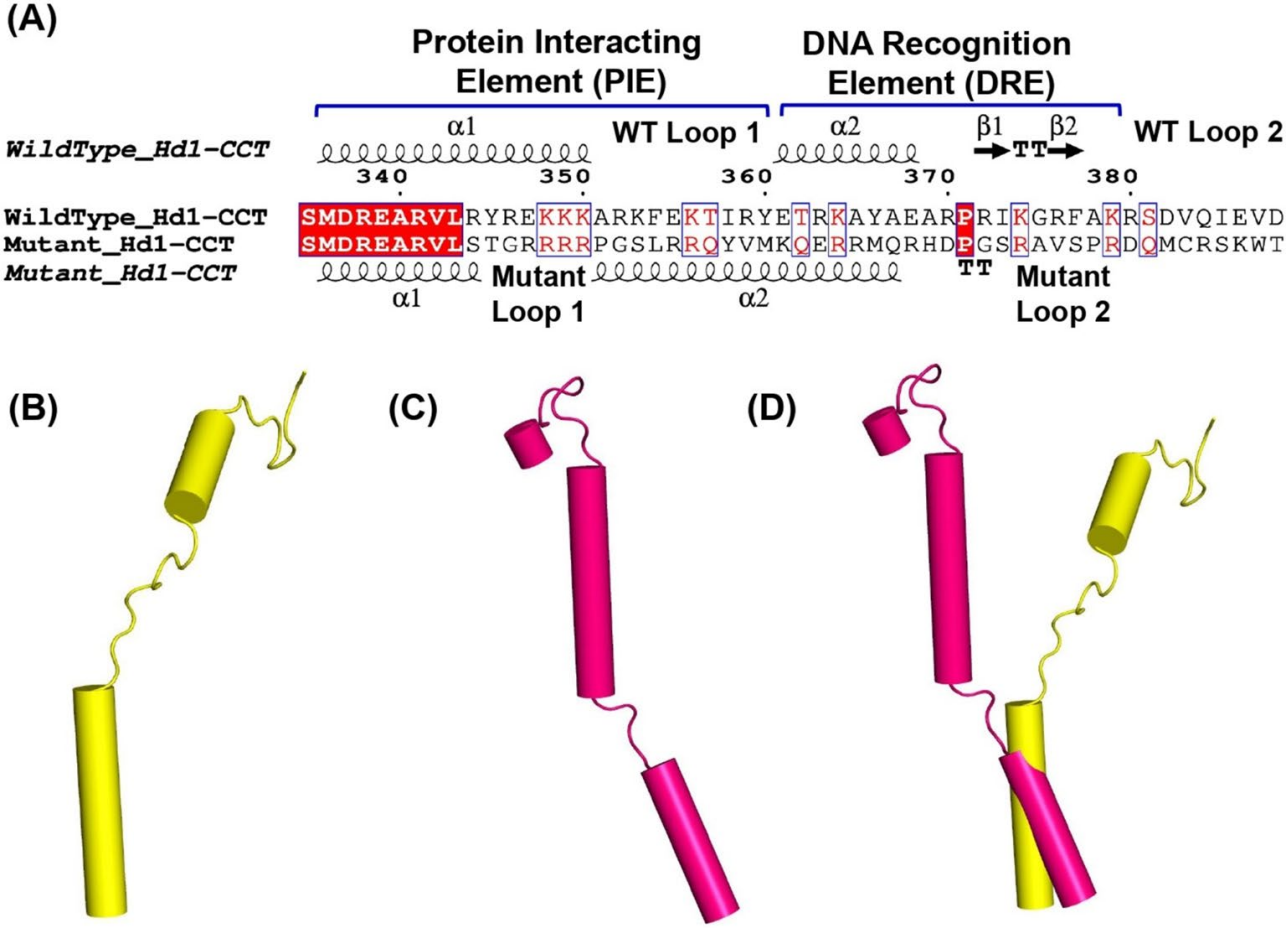
Comparison of the wild-type and mutant HD1-CCT domains. **A** Comparison of the primary sequence and secondary structural elements. **B** Predicted secondary structure elements of the wild-type HD1-CCT domain (yellow). **C** Predicted secondary structure elements of the mutant HD1-CCT domain (magenta). **D** Superposition of the wild-type (yellow) and mutant (magenta) HD1-CCT domains

A single-base deletion in exon 2 of Hd1 leads to a frameshift mutation at the C-terminus of the Hd1 protein. The C-terminus of Hd1 contains a CCT {Constant (CO), CO-LIKE (COL) and TIMING OF CAB EXPRESSION1 (TOC1)} domain. Due to this frameshift, the mutant Hd1 protein has an altered primary sequence at the C-terminus, which includes the CCT domain (Supplementary Fig. 8). To gain insight into the structural changes caused by frameshift mutations, three-dimensional models of wild-type and mutant Hd1 proteins were constructed using AlphaFold2, and only the CCT domain was used for structural analysis (Supplementary Fig. 9). The modeled structure of the wild-type CCT domain was compared to the crystal structure of the CCT domain. The very high structural similarity shows that AlphaFold can correctly predict the structure of the CCT domain of the Hd1 protein (Supplementary Fig. 9). The wild-type Hd1 protein has a typical CCT domain containing two ɑ-helices (ɑ1 and ɑ2) and 2 loops (Fig. 6 and Supplementary Fig. 9). However, the mutant Hd1 protein showed changes in secondary structural elements, as illustrated in Fig. 6.

## Discussion

Mutations are essential to evolution. Even deleterious mutations can cause evolutionary changes, especially in small populations, by removing individuals carrying adaptive alleles at other genes [32]. During domestication, loss-of-function mutations resulting in seed dormancy and shattering of wild rice have become favorable for human consumption and usage. Following domestication in the tropics and subtropics, *Oryza sativa* cultivars gradually spread to temperate and cool regions due to the loss of photoperiod sensitivity [33]. Naturally, rice flowers are under SD conditions. Heading date is a critical trait for rice diversification and domestication [34, 35] and is controlled by multiple quantitative trait loci (QTLs). Various genetic mapping approaches have mapped a large number of heading date QTLs [9, 36–50]. Hd1 is an *Arabidopsis* CONSTANS (CO) ortholog in rice that delays and promotes heading under long-day and short-day conditions, respectively [8, 51, 52]. This gene regulates panicle development and affects yield [53]. Adapting temperate japonica rice cultivars in tropical regions results in the loss of function of Hd1 alleles [54]. Hd1 was found to be a possible target of artificial selection during domestication to diversify the heading dates of rice cultivars [55]. The present study reports the isolation of a photoperiod-insensitive mutant with a loss-of-function mutation in Hd1. This particular mutant was isolated from a gamma ray-induced mutagenized population of *Kon Joha* rice, a landrace grown for its short aromatic grain in Assam, India. After verifying its true breeding nature in successive mutant generations, the mutant was found to be genetically similar to its parent but remains highly dissimilar to the existing photoperiod-insensitive *Ahu Joha* rice genotypes (Fig. 2). The distinctness of the isolated mutant from other *Ahu Joha* genotypes prompted us to further characterize the mutant at the genetic and molecular levels. In the inheritance study, two mapping populations were raised for phenotypic and genotypic segregation in the F_2_ and F_3_ generations, respectively. The segregation patterns revealed that the induced photoperiod insensitivity in *Kon Joha* is governed by a single recessive gene. The monogenic inheritance of photoperiod insensitivity was previously reported by Jones et al. [56] and Chandraratna [57]. In parallel, screening for polymorphisms of genome-wide SSR and InDel markers between a mutant and a distant parent, *Kalijeera,* detected almost 14.2% of the polymorphisms. A BSA approach in the F_2_ population of ‘PPIS mutant × *Kalijeera*' detected the association of the RM527 marker with the photoperiod insensitivity trait. RM527 is present on chromosome 6 of the rice plant. The successful screening of 40 chromosome-6-specific SSR markers (with 5.0 and 13.2 Mbp genomic intervals) revealed five additional polymorphisms. RM19725 was linked to photoperiod insensitivity in BSA. The map interval delimited by RM527 and RM19725 represented an approximately 1.73 Mbp interval (8,134,111 to 9,862,291 bp), within which the heading date 1 (Hd1) gene is present. Other photoperiod insensitivity genes, such as Hd17 (OsELF3), Hd3a and RFT (Rice flowering locus T), are also present in tandem on the proximal region of chromosome 6 (2,234,119 to 2,942,452 bp). None of the other SSR markers around these genomic boundaries of chromosome 6 showed association/linkage with photoperiod insensitivity in the isolated mutant (Supplementary Fig. 3; Supplementary Table S2). The other two photoperiod insensitivity genes, Se5/OsHY1 and OsNF-YC4/OsHAP5B, are present at the very distal end of chromosome 6, from 23,853,714 to 23,858,061 bp and 27,627,767 to 27,633,058 bp, respectively [58].

After confirming the position of the mutant photoperiod insensitivity gene in the PPIS mutant, we cloned Hd1 from both the mutant and the parent genomic DNA. A single-base deletion in exon 2 of *Hd1* results in a dysfunctional Hd1 protein. This single-base deletion causes a frameshift mutation in the C-terminus of Hd1 proteins, leading to a mutant Hd1 protein with an altered primary sequence at the C-terminus (Supplementary Fig.

8). The Hd1 protein has a zinc finger B-box domain near the N-terminus and a CCT domain (residues 323–387) near the C-terminus, followed by a single helix and a 10-residue tail. The CCT domain at the C-terminus is crucial for the binding of Hd1 to the GHD8/OsNF-YC2 dimer to form the Hd1-GHD8/OsNF-YC2 trimer, which specifically targets a CCACA nucleotide sequence within the Hd3a promoter [10]. In the crystal structure, Shen et al. [10] reported that the CCT domain folds into 2 ɑ-helices (ɑ1 and ɑ2) and 2 loops (L1 and L2) (Fig. 6 and Supplementary Fig. 9). Helix ɑ1 (residues 335–351) and loop1 (residues 352–360) together form a protein-interacting element (PIE, residues 335–360) and interact with the GHD8/OsNF-YC2 dimer, yielding a functional trimer (Fig. 6 and Supplementary Fig. 9 & 10). Helix ɑ2 (residues 361–369) and loop2 (residues 370–379) together form the DNA recognition element (DRE, residues 361–379) of CCT, which anchors into the minor groove of the 'CCACA' box (Fig. 6 and Supplementary Fig. 9).

The crystal structure of the Hd1^CCT^-GHD8/OsNF-YC2 trimer shows that residues R341, R344, Y345, R346, and K348 from ɑ1 and R352, K353, R359 and Y360 from loop1 regions form hydrogen bonds with different residues of GHD8 and OsNF-YC2 (Supplementary Figs. 11 & 12). Among these residues, R338 and Y345 in the PIE are crucial for the binding of Hd1 to the GHD8/OsNF-YC2 dimer, and R338A and Y345A mutations abrogate the formation of the HD1/GHD8/OsNF-YC2 trimer [10]. These residues are also conserved in the HD1 homolog in *Arabidopsis* and participate in similar interactions [59]. Hd1^CCT^ residues involved in the interactions with GHD8 and OsNF-YC2 are primarily positively charged residues that interact with negatively charged residues from GHD8 and OsNF-YC2 in a zipper-like arrangement (Supplementary Fig. 13). The structure of the HD1/GHD8/OsNF-YC2 trimer-DNA complex also suggested that loop 1 acts as a flexible linker between the ɑ1 and ɑ2 helices and helps in the proper positioning of the ɑ2 helix in the minor groove of the 'CCACA' box. Therefore, both the positively charged residues and the nature of the secondary structural elements needed to properly orient these residues are crucial for binding the CCT domain to the GHD8/OsNF-YC2 dimer.

A single-base deletion in exon 2 of Hd1 leads to a frameshift mutation in the middle of the CCT domain, resulting in a mutant Hd1 protein with an altered protein sequence at the C-terminal region (Fig. 6). Since the mutation site is located in the middle of the PIE (Protein interaction element)  of the CCT domain, it changes the primary sequence and the secondary structure of the latter part of the PIE and completely alters the sequence and structure of the DRE (DNA recognition element) of the CCT (Fig. 6 and Supplementary Fig. 9). The mutant CCT domain likely folds into a helix and loop structure. However, computational modeling suggested distinct differences in the wild-type and mutant CCT domains. In the mutant CCT domain, the crucial ɑ1 helix is truncated, and the C-terminal part of the ɑ1 helix becomes unstructured.

Similarly, loop 1 regions become helical and form a more prominent helix in continuity with helix ɑ2 (Fig. 6). At the residue level, in the PIE of the mutant CCT domain, the crucial tyrosine residue (Y345) is replaced with threonine, and all the other essential residues, such as R346 and K348 from ɑ1 and R352, K353, R359 and Y360 of the loop region, are also altered (Fig. 6). Since both positively charged residues and specific secondary structures are required for the interaction of the CCT domain with the GHD8/OsNF-YC2 dimer, these alterations are likely to cause the loss of this crucial interaction. Therefore, this altered CCT domain of Hd1 is unlikely to bind to the GHD8/OsNF-YC2 dimer to form a repressive protein trimer complex. This particular CCT domain is essential for yielding a histone-like structure along with the DTH8 and NF-Y proteins via interaction of the promoter element of the florigen gene containing the CCACA element [10]. The changes in the primary and secondary structures of the CCT domain in the mutant Hd1 protein are likely to compromise the interaction with the CCACA element that leads to relaxed repression of Hd3a and RFT genes under long-day conditions. In the absence of the trimeric complex interaction with the CCACA box of the Hd3a promoter, the interaction is also likely to be affected, leading to the observed phenotype.

## Conclusion

The cultivation of Assam's aromatic *Joha* rice, a unique rice class famous for its aroma, taste, and nutritional benefits and high market prices, is decreasing to 5% of the winter rice area in the state, mainly due to poor productivity, long duration, limited seasonal adaptability, and high production costs. Short-*grain* aromatic *Joha* rice is mostly photoperiod sensitive and least responsive to applied fertilizers. These fragrant low- or no-input rice cultivars, which are organic by default, have evolved to thrive in rainfed shallow lowland rice ecosystems. Here, we report a novel mutant allele of heading date 1 (Hd1) conferring photoperiod insensitivity in the famous *Joha* rice variety of Assam. This mutant allele, when adopted, could lead to significant increases in yield, reducing production costs and increasing profits for farmers. The deciphered genetic basis of this mutant allele, which involves a mutation in the Hd1 gene, has underscored the urgent need for a structured breeding programme in high-value *Joha* rice, highlighting the necessity of immediate action.

## Supplementary Information

Supplementary Material 1.

Supplementary Material 2.

## Data Availability

All data generated or analyzed during this study are included in this published article and its supplementary information files.
