# Supplementary material for: A single-base deletion in exon 2 of Hd1 delineates monogenic recessive photoperiod insensitivity in aromatic Joha rice: a novel allele for seasonal adaptability
Source: Biol Res. 2024 Nov 30;57:94. doi: 10.1186/s40659-024-00553-7 (PMC11607960; doi:10.1186/s40659-024-00553-7)
Supplement: Supplementary file 1 — Supplementary Material 1. [file 40659_2024_553_MOESM1_ESM.docx]

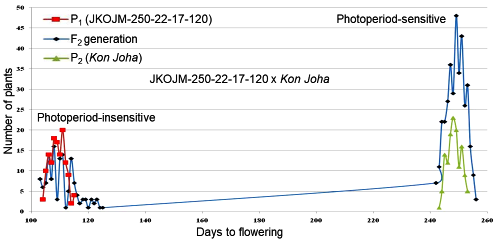


Supplementary Figure 1 Bimodal distribution of F_2_ individuals for their responses to photoperiod.


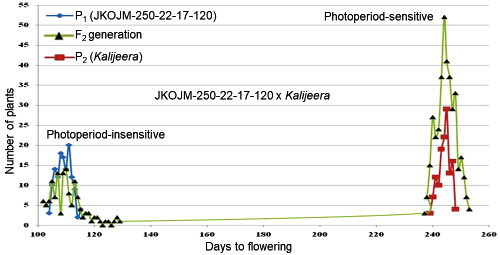


Supplementary Figure 2 Bimodal distribution of F_2_ individuals for their responses to photoperiod.


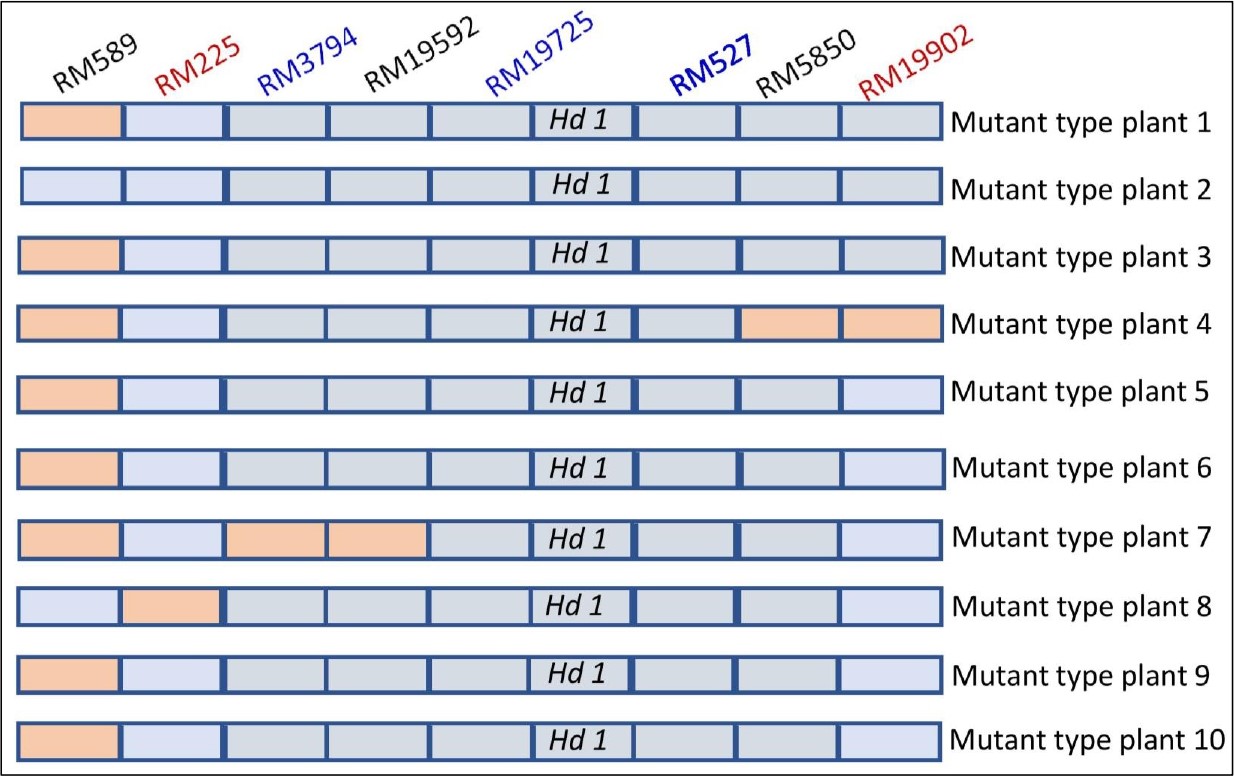


Supplementary Figure 3 Pictorial representation of allelic diversity of chromosome 6-specific SSR markers in 10 individual F _2_  mutant plants of ‘Mutant x *Kalijeera*’.


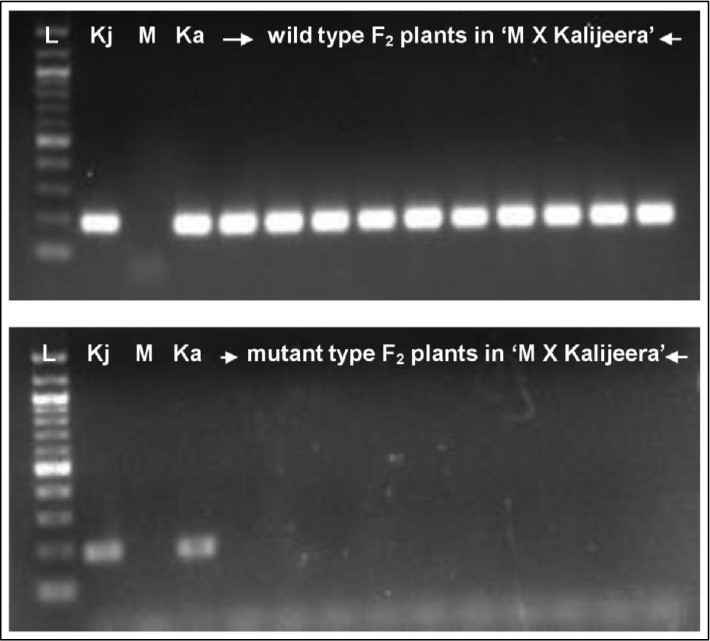


Supplementary Figure 4 PCR of parental allele-specific S NAP markers.


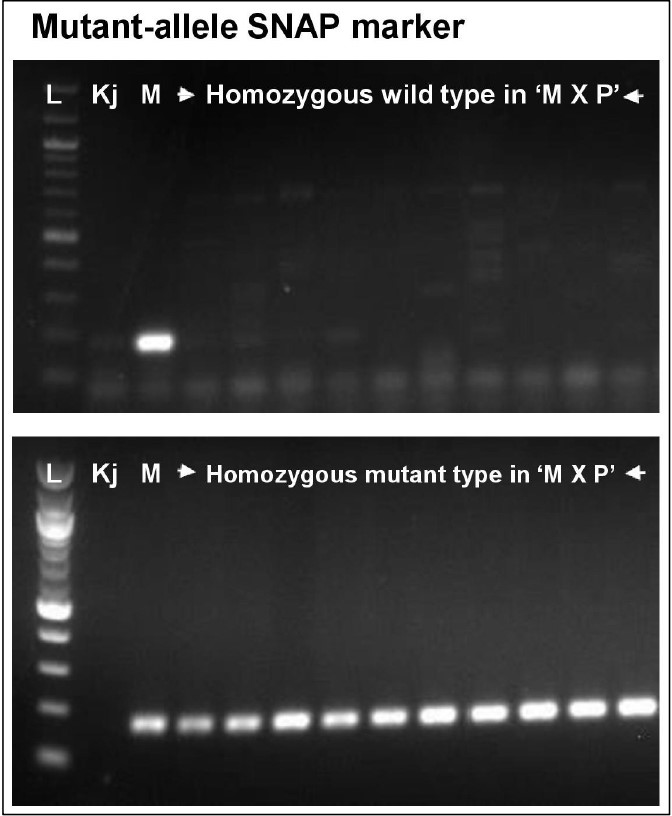


Supplementary Figure 5 PCR of the mutant allele-specific SNAP marker.


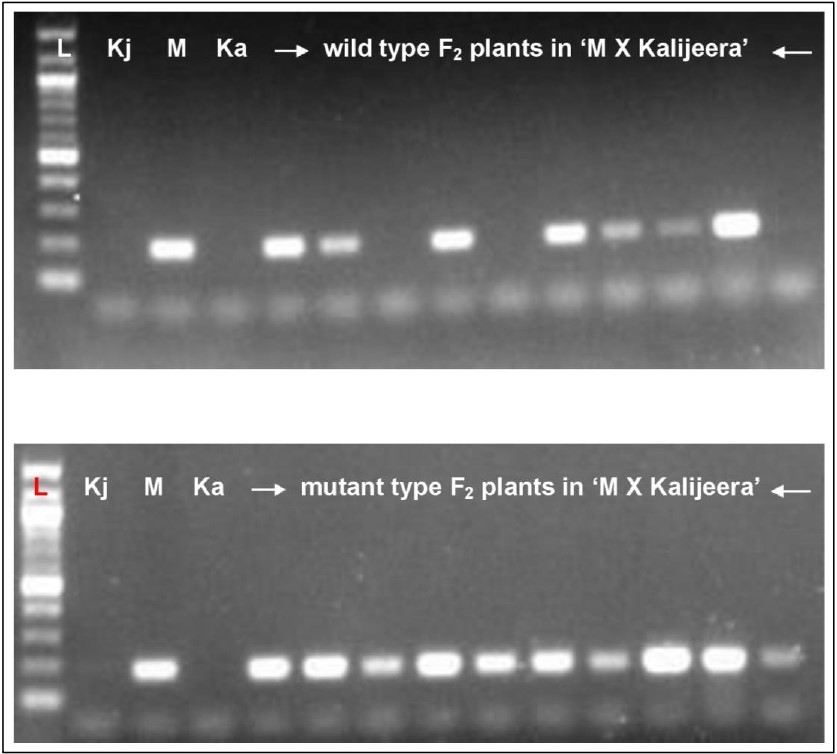


Supplementary Figure 6 Mutant allele-specific SNAP reaction in ‘PISM x *Kalijeera*’ F_2_ individuals.


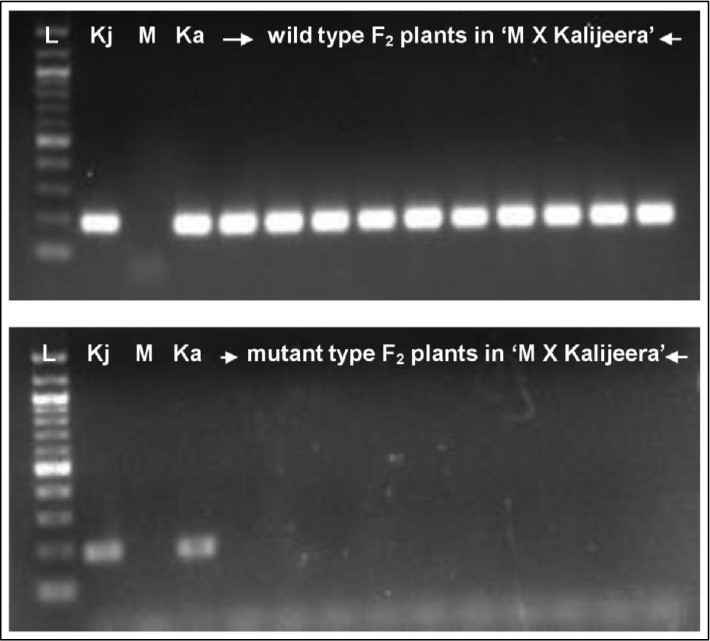


Supplementary Figure 7 Parental allele-specific SNAP reaction in ‘PISM x *Kalijeera*’ F_2_ individuals.


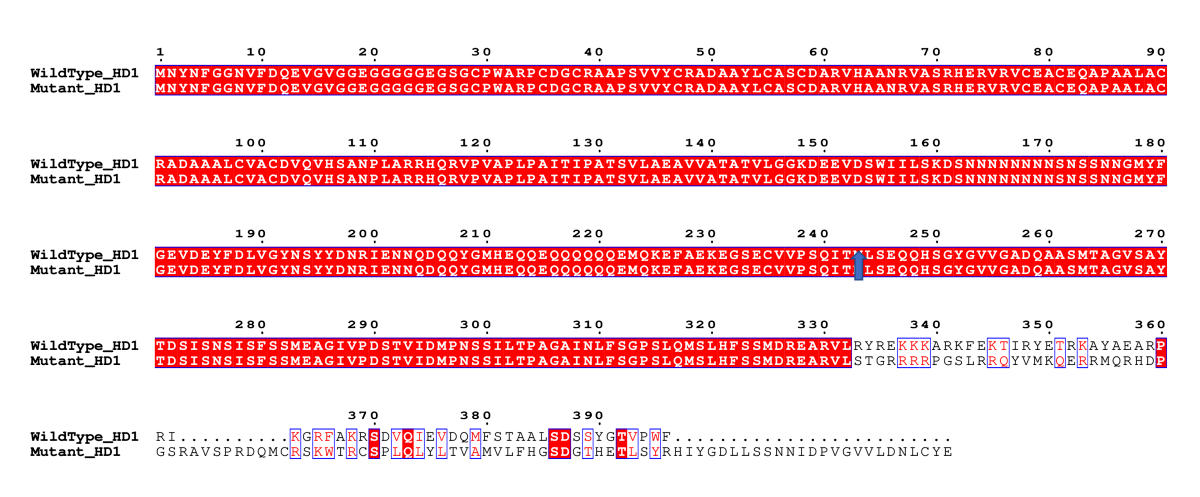


Supplementary Figure 8 Sequence alignment of wild-type and mutant HD1 proteins. Mutation site is marked with arrow.


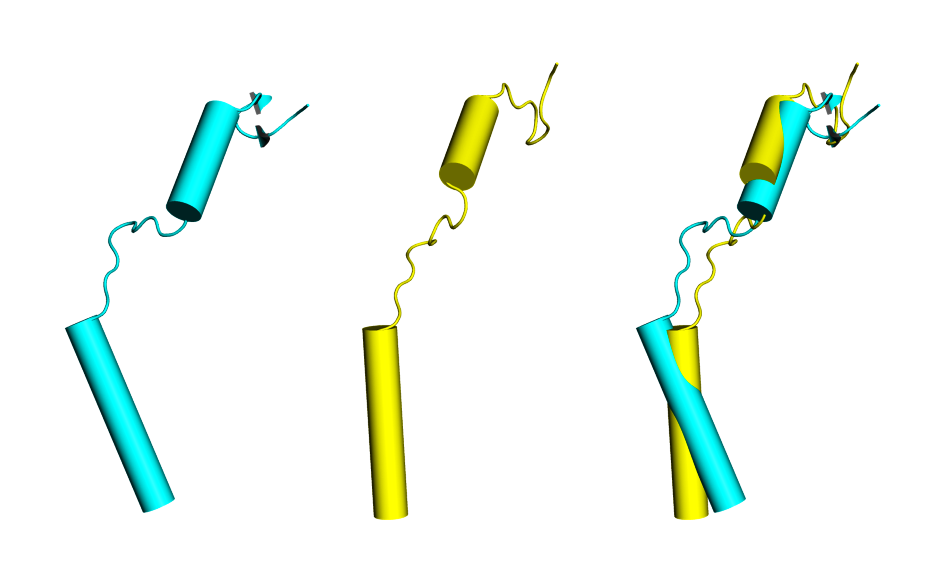


Supplementary Figure 9 Secondary structure elements of HD1-CCT domain (cyan) (PDB ID 7C9O). Predicted Secondary structure elements of HD1-CCT domain using alphafold (yellow). Superposition of cyrstal structure and modelled structures of HD1-CCT domain showing accuracy of alphafold’s prediction.


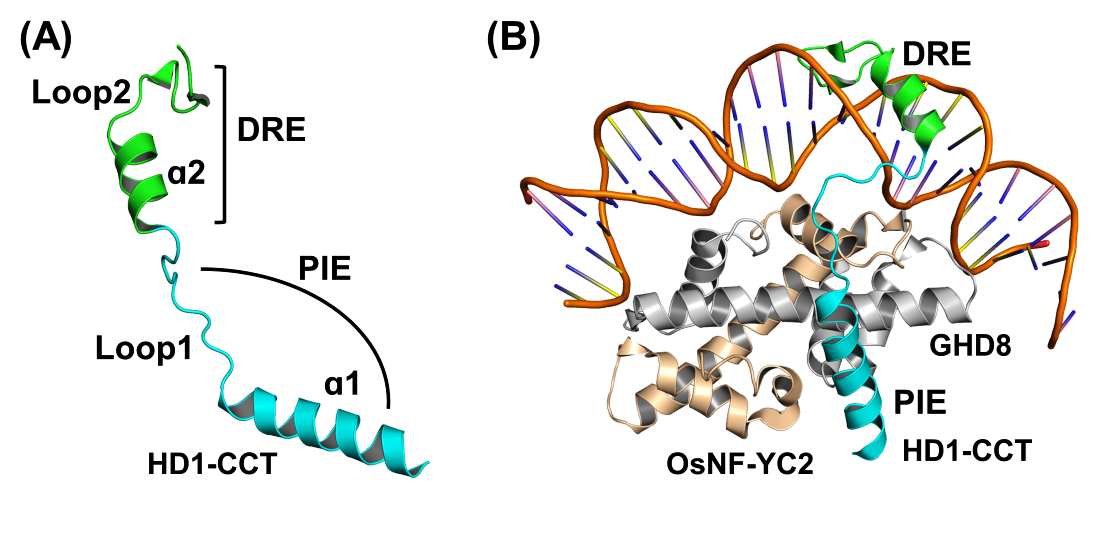


Supplementary Figure 10 (A) Cartoon representation of tertiary structure of HD1-CCT domain (PDB Id 7C9O). Protein interaction element (PIE) is shown in cyan and DNA recognition element (DRE) is in green. (B) Interaction of HD1-CCT domain in the DNA-bound HD1-GHD8/OsNF-YC2 trimer (PDB Id 7C9O). Protein interaction element (PIE) is shown in cyan and DNA recognition element (DRE) is in green. GHD8 is shown in grey while OsNF is in wheatish color.


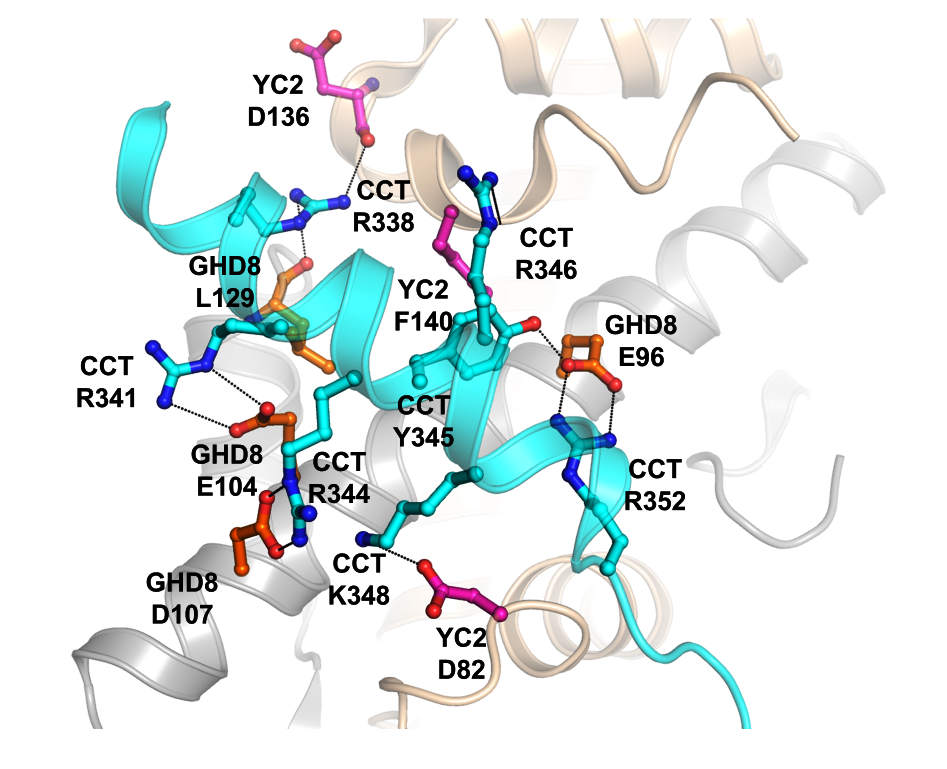


Supplementary Figure 11 Interactions between ɑ1 helix of protein interaction element (PIE) of HD1-CCT domain (cyan) with residues from GHD8 (grey)/OsNF-YC2 (wheatish). Residue numbering is based on PDB id 7C9O.


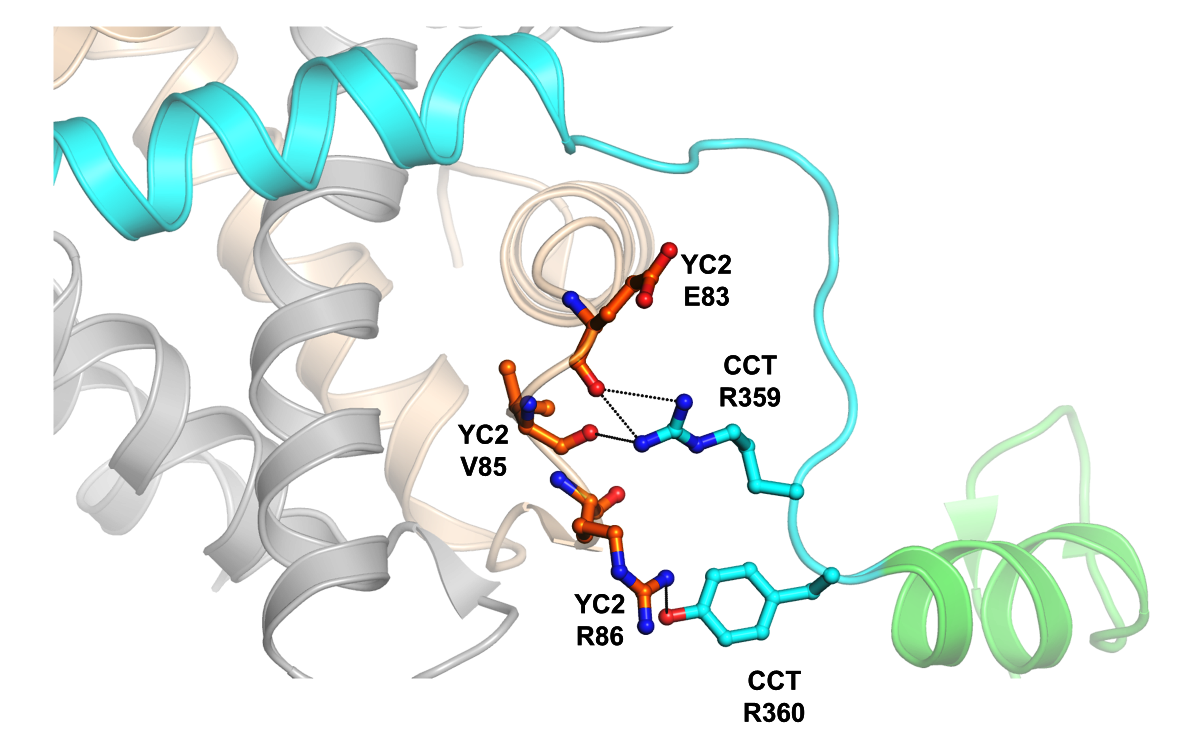


Supplementary Figure 12 Interactions between loop1 of protein interaction element (PIE) of HD1-CCT domain (cyan) with residues from OsNF-YC2 (wheatish). GHD8 is also shown in grey. Residue numbering is based on PDB id 7C9O.


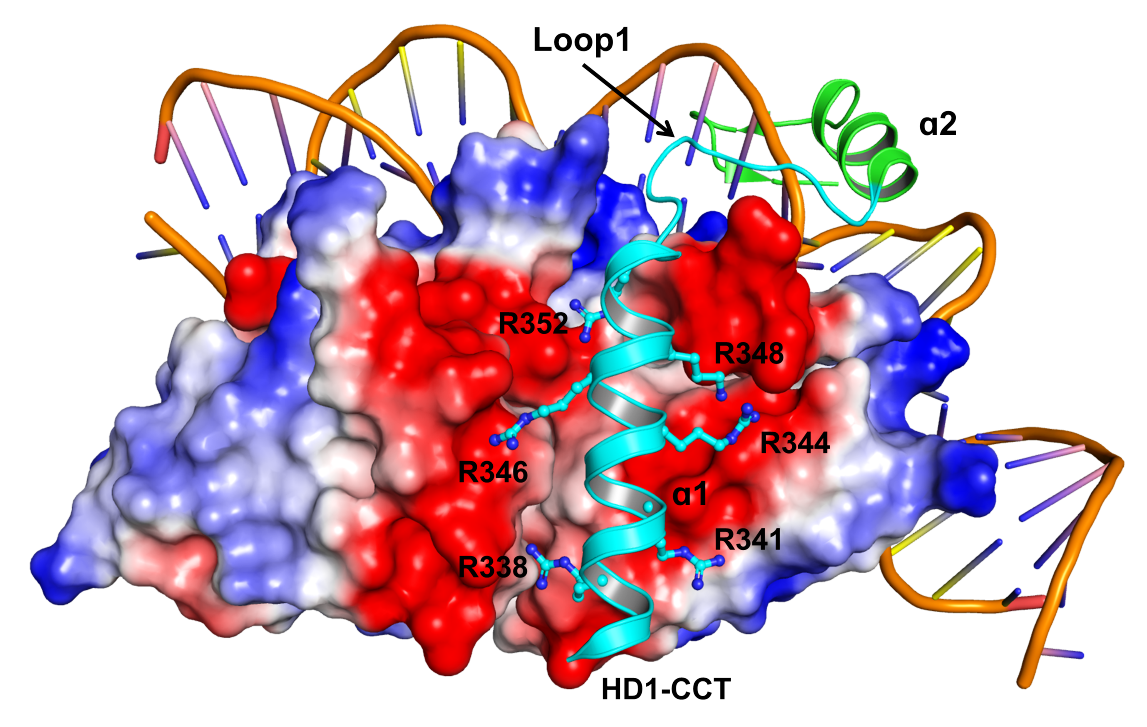


Supplementary Figure 13 Zipper like arrangement of positively charged residues from ɑ1 helix of protein interaction element (PIE) of HD1-CCT domain interacting with the negatively charged residues from GHD8/OsNF-YC2. Residue numbering is based on PDB id 7C9O.
