# Supplementary material for: A single-base deletion in exon 2 of Hd1 delineates monogenic recessive photoperiod insensitivity in aromatic Joha rice: a novel allele for seasonal adaptability
Source: Biol Res. 2024 Nov 30;57:94. doi: 10.1186/s40659-024-00553-7 (PMC11607960; doi:10.1186/s40659-024-00553-7)
Supplement: Supplementary file 2 — Supplementary Material 2. [file 40659_2024_553_MOESM2_ESM.pdf]

Supplementary Table S1 Details of SSR and InDel markers used for detection of genomic integrity of mutant and gene tagging.

Sl. No.	Marker Name	Forward Primer Sequence (5'-3')	Reverse Primer Sequence (5'-3')	Product size	Repeat motif	Chr. No.	Marker Type	PPIS mutant vs *Kalijeera*
1	RM600	AAACGTGTGTTAGCCTGTTAGG	CATATGCTAGTGGTGCTAGCG	220	(TTA)19	1	SSR	Polymorphic
2	RM129	TCTCTCCGGAGCCAAGGCGAGG	CGAGCCACGACGCGATGTACCC	205	(CGG)8	1	SSR	
3	RM11943	CTTGTTCGAGGACGAAGATAGGG	CCAGTTTACCAGGGTCGAAACC	76	(GA)11	1	SSR	
4	RM292*	ACTGCTGTTGCGAAACGC	TGCAGCAAATCAAGCTGGAA	159	(GT)10-6-(TGA)2TGT(TGA)4	1	SSR	
5	RM212	CCACTTTCAGCTACTACCAG	CACCCATTTGTCTCTCATTATG	136	(CT)24	1	SSR	
6	RM237*	CAAATCCCGACTGCTGTCC	TGGGAAGAGAGCACTACAGC	191	(CT)18	1	SSR	
7	RM486	CCCCCCTCTCTCTCTCTCTC	TAGCCACATCAACAGCTTGC	104	(CT)14	1	SSR	
8	RM3602	TGAAAAGCCACTCAGATGCG	TGGTGAAAGGGTCAGAACTG	120	(GA)13	1	SSR	Polymorphic
9	RM472	CCATGGCCTGAGAGAGAGAG	AGCTAAATGGCCATACGGTG	296	(GA)21	1	SSR	
10	RM3521	CCTTCGAGTGCATGTCCC	GAACACGAGGAGAATGAGCC	209	(CT)31	1	SSR	Polymorphic
11	RM431*	TCCTGCGAACTGAAGAGTTG	AGAGCAAAACCCTGGTTCAC	251	(AG)16	1	SSR	
12	RM319	ATCAAGGTACCTAGACCACCAC	TCCTGGTGCAGCTATGTCTG	134	(GT)10	1	SSR	
13	RM12023	TGCGTACCTCTGCTCCTCTCTGC	GACGAAGCCGACCAAGTGAAGC	93	(TCG)8	1	SSR	
14	RM104*	GGAAGAGGAGAGAAAGATGTGTGTCG	TCAACAGACACACCGCCACCGC	222	(GA)9	1	SSR	
15	RM1141	TGCATTGCAGAGAGCTCTTG	CAGGGCTTTGTAAGAGGTGC	100	(AG)12	1	SSR	
16	RM259*	TGGAGTTTGAGAGGAGGG	CTTGTTGCATGGTGCCATGT	162	(CT)17	1	SSR	
17	RM3604	ATGTCAGACTCCGATCTGGG	TCTTGACCTTACCACCAGGC	153	(GA)13	1	SSR	Polymorphic
18	RM315	GAGGTACTTCCTCCGTTTCAC	AGTCAGCTCACTGTGCAGTG	133	(AT)4(GT)10	1	SSR	
19	RM3148	GACTATTGCTCGAACACTTTG	TTGTCTGCTTTGGTATTTGC	166	(CA)20	1	SSR	Polymorphic
20	RM513*	TCTAGTGGCCTCAAAAAGGG	GCAACGAAATCATCCCTAGC	262	(TC)11	1	SSR	
21	RM12091	CTGCAAATGCACAGGAATCAGG	TCCTCTCGCCTTTCTTTCTCTCC	142	(AG)31	1	SSR	Polymorphic
22	RM5638	GGCTTCCTCATCGCCATC	CTGAGCAGCATTCCAGTCTG	203	(AAG)13	1	SSR	
23	RM297	TCTTTGGAGGCGAGCTGAG	CGAAGGGTACATCTGCTTAG	148	(GA)13	1	SSR	
24	RM3825	AAAGCCCCCAAAAGCAGTAC	GTGAAACTCTGGGGTGTTCG	147	(GA)21	1	SSR	
25	RM9	GGTGCCATTGTCGTCCTC	ACGGCCCTCATCACCTTC	136	(GA)15GT(GA)2	1	SSR	
26	RM488*	CAGCTAGGGTTTTGAGGCTG	TAGCAACAACCAGCGTATGC	177	(GA)17	1	SSR	
27	RM24	GAAGTGTGATCACTGTAACC	TACAGTGGACGGCGAAGTCG	192	(GA)5	1	SSR	
28	RM14	CCGAGGAGAGGAGTTCGAC	GTGCCAATTTCCTCGAAAAA	191	(GA)18	1	SSR	
29	RM243	GATCTGCAGACTGCAGTTGC	AGCTGCAACGATGTTGTCC	116	(CT)18	1	SSR	
30	RM12146	AGTATGCCCTGCCCACTACACTAGG	CAGCGAATGGCAAGAGCAACC	99	(AG)11	1	SSR	
31	RM495*	AATCCAAGGTGCAGAGATGG	CAACGATGACGAACACAACC	159	(CTG)7	1	SSR	

32	RM1*	GCGAAAACACAATGCAAAAA	GCGTTGGTTGGACCTGAC	113	(GA)26	1	SSR	
33	RM283*	GTCTACATGTACCCTTGTTGGG	CGGCATGAGAGTCTGTGATG	151	(GA)18	1	SSR	
34	RM312*	GTATGCATATTTGATAAGAG	AAGTCACCGAGTTTACCTTC	97	(ATTT)4(GT)9	1	SSR	
35	RM5*	TGCAACTTCTAGCTGCTCGA	GCATCCGATCTTGATGGG	113	(GA)14	1	SSR	
36	RM272	AATTGGTAGAGAGGGGAGAG	ACATGCCATTAGAGTCAGGC	119	(GA)9	1	SSR	
37	RM005	TGCAACTTCTAGCTGCTCGA	GCATCCGATCTTGATGGG	113	(GA)14	1	SSR	
38	RM23	CATTGGAGTGGAGGCTGG	GTCAGGCTTCTGCCATTCTC	145	(GA)15	1	SSR	
39	R1M7*	ATTCCTGGTTCTACATTACTTA	CGCCTCACTAGAATATCGGA			1	InDel	Polymorphic
40	R1M30*	AAGGGGCCCTAATTTATCTAG	TGTTTACTTTGTTCTTGGACTG			1	InDel	
41	R1M37*	ATAGTTCGCCATCGTCAT	ACACGCCATAGCAAGGAA			1	InDel	
42	R1M47*	AATAGAATTACTGATGAAACCTTA	GCCCGTTACCGCTTATGT			1	InDel	
43	RM530*	GCACTGACCACGACTGTTTG	ACCGTAACCCGGATCTATCC	161	(GA)23	2	SSR	
44	RM573*	CCAGCCTTTGCTCCAAGTAC	TCTTCTTCCCTGGACCACAC	201	(GA)11	2	SSR	
45	RM2634	GATTGAAAATTAGAGTTTGCAC	TGCCGAGATTTAGTCAACTA	154	(AT)31	2	SSR	
46	RM279*	GCGGGAGAGGGATCTCCT	GGCTAGGAGTTAACCTCGCG	174	(GA)16	2	SSR	
47	RM341	CAAGAAACCTCAATCCGAGC	CTCCTCCCGATCCCAATC	172	(CTT)20	2	SSR	
48	RM262	CATTCCGTCTCGGCTCAACT	CAGAGCAAGGTGGCTTGC	154	(CT)16	2	SSR	
49	RM475	CCTCACGATTTTCCTCCAAC	ACGGTGGGATTAGACTGTGC	235	(TATC)8	2	SSR	
50	RM207	CCATTCGTGAGAAGATCTGA	CACCTCATCCTCGTAACGCC	118	(CT)25	2	SSR	
51	RM138	AGCGCAACAACCAATCCATCCG	AAGAAGCTGCCTTTGACGCTATGG	233	(GT)14	2	SSR	
52	RM12811	ACAGTACACTAGGACATGGTTACG	GTCTAATTGCAGAATGCAGACC	254	(AT)35	2	SSR	
53	RM327*	CTACTCCTCTGTCCCTCCTCTC	CCAGCTAGACACAATCGAGC	213	(CAT)11(CTT)5	2	SSR	
54	RM301*	TTACTCTTTGTGTGTGTGTGAG	CTACGACACGTCATAGATGACC	153	(GT)5G2(GT)8T2(GT)3	2	SSR	
55	RM236	GCGCTGGTGGAAAATGAG	GGCATCCCTCTTTGATTCCTC	191	(CT)18	2	SSR	
56	RM1789	GGAAATGTACAGATGTGTGG	CAATCTCGCAATTTTTCATA	125	(AT)16	2	SSR	
57	RM526	CCCAAGCAATACGTCCCTAG	ACCTGGTCATGACAAGGAGG	240	(TAAT)5	2	SSR	
58	RM203	CCTATCCCATTAGCCAAACATTGC	GATTTACCTCGACGCCAACCTG	203	(AT)21	2	SSR	
59	RM233*	CCAAATGAACCTACATGTTG	GCATTGCAGACAGCTATTGA	162	(CT)20	2	SSR	
60	RM253	TCCTTCAAGAGTGCAAAACC	GCATTGTCATGTCGAAGCC	141	(GA)25	2	SSR	
61	RM5404	GGCCATCCATCTCCTGTATG	GACACACACAGGGTTGGTTG	125	(TC)15	2	SSR	
62	RM106	CGTCTTCATCATCGTCGCCCCG	GGCCCATCCCGTCGTGGATCTC	297	(GAA)5	2	SSR	
63	RM174	AGCGACGCCAAGACAAGTCGGG	TCCACGTCGATCGACACGACGG	208	(AGG)7(GA)10	2	SSR	
64	RM211	CCGATCTCATCAACCAACTG	CTTCACGAGGATCTCAAAGG	161	(TC)3A(TC)18	2	SSR	
65	RM250	GGTTCAAACCAAGCTGATCA	GATGAAGGCCTTCCACGCAG	153	(CT)17	2	SSR	
66	RM53	ACGTCTCGACGCATCAATGG	CACAAGAACTTCCTCGGTAC	182	(GA)14	2	SSR	

67	RM452*	CTGATCGAGAGCGTTAAGGG	GGGATCAAACCACGTTTCTG	209	(GTC)9	2	SSR	
68	RM12460	TGGCACTACAGTGACAACAAACC	AGGGACTTTATCCAAAGGACACG	168	(TC)16	2	SSR	
69	RM240	CCTTAATGGGTAGTGTGCAC	TGTAACCATTCCTTCCATCC	132	(CT)21	2	SSR	
70	RM6374	TGAGGACGCTGATTGTCAAC	GCTGCCCCTATTATTTCACC	127	(GAA)16	2	SSR	Polymorphic
71	RM13600	GGTTAACCTTTCTCGCTCTTTGG	ATGATCCAAACCCACTGTCTTCC	122	(AG)11	2	SSR	
72	RM1367	GTGTGTACGTAGGATCGGAG	TGCTACTCCTAGCTGCTACC	159	(AG)27	2	SSR	
73	RM521	TTCCCTTATTCCTGCTCTCC	GGGATTTGCAGTGAGCTAGC	260	(TC)14	2	SSR	
74	RM12868	ACTCATCAACATCGCGAAACAGC	CTGACTCCGACAACAGCTATGAACC	143	(ATC)7	2	SSR	
75	RM3549	AGACGACAAACACCTGCCTC	CAAACACAAACGCAGCCTC	168	(GA)12	2	SSR	
76	RM555	TTGGATCAGCCAAAGGAGAC	CAGCATTGTGGCATGGATAC	223	(AG)11	2	SSR	
77	RM109	GCCGCCGGAGAGGGAGAGAGAG	CCCCGACGGGATCTCCATCGTC	97	(AG)16	2	SSR	
78	RM154*	ACCCTCTCCGCCTCGCCTCCTC	CTCCTCCTCCTGCGACCGCTCC	183	(GA)21	2	SSR	
79	RM6	GTCCCCTCCACCCAATTC	TCGTCTACTGTTGGCTGCAC	163	(AG)16	2	SSR	
80	RM263	CCCAGGCTAGCTCATGAACC	GCTACGTTTGAGCTACCACG	199	(CT)34	2	SSR	
81	OSR17	GCTGGTTGATTCAGCTAGTC	GCCTCGTTGTCGTTCCACAC	100	(AATT)n	2	SSR	
82	RM12569	GCTCATCATCATCATCGCAGTGG	ATCCATGTGGCAGACACACTTGC	129	(AG)10	2	SSR	
83	RM450*	AAACCACAGTAGTACGCCGG	TCCATCCACATCTCCCTCTC	143	(AG)17	2	SSR	
84	RM3316*	TTCGACGATTCTGTACACGC	CATGATCCCAAATGCATGGG	207	(CT)14	2	SSR	
85	RM3789*	ATTAAGGGCAGGGGCATATC	CATTGACTGGTGTGGTCAGG	121	(GA)19	2	SSR	Polymorphic
86	RM8030	CTTACATTATGAAACGGATG	ATAACAAAACCACACTTTGA	128	(AT)24	2	SSR	
87	R2M10*	CCCAGTCTGCTGCCATCT	GAATGTATTTCAGTTCCAGTAAG			2	InDel	
88	R2M24*	GGGCAACAACGGCTCTG	AGGGAATAAGGCGATACGG			2	InDel	
89	R2M26*	GCAGCAAAGTGCGGAGTA	CAGGTGAATTGCCAATTT			2	InDel	
90	R2M37*	ACTGTTACCCAAACGCTA	ACGTGCACCTACTACAGAAA			2	InDel	Polymorphic
91	R2M50*	CCTGAAGGAAATGATAGCAATAG	GTTTTGTATGCTCTTCACTTGTC			2	InDel	
92	RM22	GGTTTGGGAGCCCATAATCT	CTGGGCTTCTTTCACTCGTC	194	(GA)22	3	SSR	
93	RM520	AGGAGCAAGAAAAGTTCCCC	GCCAATGTGTGACGCAATAG	247	(AG)10	3	SSR	
94	RM15078	TCTTAAGCTCCTCATCGTTTGC	CCTTGCGATGATTAATGTGC	374	(TATG)6	3	SSR	
95	RM231*	CCAGATTATTTCCTGAGGTC	CACTTGCATAGTTCTGCATTG	182	(CT)16	3	SSR	
96	RM1352	ACGAGTTGTACTCTGGTTGC	TCTCGGTTTTTATCTTGCTG	219	(AG)23	3	SSR	
97	RM7332	ACACTGTACACCACACTTCAGC	CAGGGAAATGACACTGTCCC	205	(ACAT)11	3	SSR	
98	RM251	GAATGGCAATGGCGCTAG	ATGCGGTTCAAGATTCGATC	147	(CT)29	3	SSR	
99	RM85*	CCAAAGATGAAACCTGGATTG	GCACAAGGTGAGCAGTCC	107	(TGG)5(TCT)12	3	SSR	
100	RM1038	TGGTTCGATTCGGATTTC	AAGCTATTCACAAGCAGCTC	172	(AC)14	3	SSR	Polymorphic
101	RM523*	AAGGCATTGCAGCTAGAAGC	GCACTTGGGAGGTTTGCTAG	148	(TC)14	3	SSR	

102	RM55*	CCGTCGCCGTAGTAGAGAAG	TCCCGGTTATTTTAAGGCG	226	(GA)17	3	SSR	
103	RM232	CCGGTATCCTTCGATATTGC	CCGACTTTTCCTCCTGACG	158	(CT)24	3	SSR	
104	RM15291	TACCATCCAACCATCCAAGAACC	CGGTACACCGCTTTAACCTGTCC	153	(TC)10	3	SSR	
105	RM416*	GGGAGTTAGGGTTTTGGAGC	TCCAGTTTCACACTGCTTCG	114	(GA)9	3	SSR	
106	RM1256	ACGCGAAGCAACGGAGATAG	CTAGCCTCGATGCGAAAAAC	148	(AG)16	3	SSR	
107	RM156*	GCCGCACCCTCACTCCCTCCTC	TCTTGCCGGAGCGCTTGAGGTG	160	(CGG)8	3	SSR	
108	RM132*	ATCTTGTTGTTTCGGCGGCGGC	CATGGCGAGAATGCCCACGTCC	83	(CGG)8	3	SSR	
109	RM426	ATGAGATGAGTTCAAGGCCC	AACTCTGTACCTCCATCGCC	150	(CA)10	3	SSR	Polymorphic
110	RM569	GACATTCTCGCTTGCTCCTC	TGTCCCCTCTAAAACCCTCC	175	(CT)16	3	SSR	
111	RM517	GGCTTACTGGCTTCGATTTG	CGTCTCCTTTGGTTAGTGCC	266	(CT)15	3	SSR	Polymorphic
112	RM6349	CGTCCACTCGTGACAATGAC	TGATCTCCTCCTCCTCCTCC	97	(GAA)9	3	SSR	Polymorphic
113	RM15956	CAATTTCAGACATCCACCATGC	CTCGTCCCATGCACTAACCTAGC	279	(TA)10	3	SSR	Polymorphic
114	RM168	TGCTGCTTGCCTGCTTCCTTT	GAAACGAATCAATCCACGGC	116	T15(GT)14	3	SSR	
115	RM186	TCCTCCATCTCCTCCGCTCCCG	GGGCGTGGTGGCCTTCTTCGTC	124	(CGG)5	3	SSR	
116	OSR13*	CATTTGTGCGTCACGGAGTA	AGCCACAGCGCCCATCTCTC	0	(GA)n	3	SSR	
117	RM489*	ACTTGAGACGATCGGACACC	TCACCCATGGATGTTGTCAG	271	(ATA)8	3	SSR	
118	RM5548	GGTGCAGAGTGATGCAATTC	AACATTAGGGATGAGGCTGG	144	(TG)14	3	SSR	
119	RM571	GGAGGTGAAAGCGAATCATG	CCTGCTGCTCTTTCATCAGC	188	(GT)11(AG)13	3	SSR	
120	RM1373	TGCTATACCCAAATGTCCAAGC	ATCTTCTGAGTGCTGCCAAGC	400	(AG)34	3	SSR	Polymorphic
121	RM60	AGTCCCATGTTCCACTTCCG	ATGGCTACTGCCTGTACTAC	165	(AATT)5AATCT(AATT)	3	SSR	
122	RM468	CCCTTCCTTGTTGTGGCTAC	TGATTTCTGAGAGCCAACCC	265	(TAT)8	3	SSR	
123	RM5442	AGGAGACAGGAAAGCCTTCC	CGAGTCGACCAGGCTAGAAC	172	(TC)17	3	SSR	
124	RM338*	CACAGGAGCAGGAGAAGAGC	GGCAAACCGATCACTCAGTC	183	(CTT)6	3	SSR	
125	RM514*	AGATTGATCTCCCATTCCCC	CACGAGCATATTACTAGTGG	259	(AC)12	3	SSR	
126	RM7000	CCCTTCTTTTCAACTGAATA	TTGTAACAATGAACTCGTTC	138	(TTG)10	3	SSR	Polymorphic
127	RM411	ACACCAACTCTTGCCTGCAT	TGAAGCAAAAACATGGCTAGG	110	(GTT)7	3	SSR	
128	RM422	TTCAACCTGCATCCGCTC	CCATCCAAATCAGCAACAGC	385	(AG)30	3	SSR	
129	RM3894	TATGCTCTCTCCTTCAGGCC	CTTACCAACTCCGCACTTGC	201	(GT)15	3	SSR	
130	RM15449	AGACTAATGGGTTTCTGTCAGC	TTTATTCGGCTCTCACTCTGG	469	(TC)11	3	SSR	
131	RM5474	AAAGTGTTGGTGAGCATAGC	TTTGTGTTTGGAGAGACGAG	155	(TC)21	3	SSR	
132	RM282	CTGTGTCGAAAGGCTGCAC	CAGTCCTGTGTTGCAGCAAG	136	(GA)15	3	SSR	
133	RM3867*	TTGACTGGAACATCGAGCTC	ATCCCCTCTACACCGTACCC	120	(GA)30	3	SSR	Polymorphic
134	RM6970*	TCGCTTGTGTTTTCTGGGTC	TGGAGAATTTGGAGGCTGC	113	(TTC)12	3	SSR	
135	RM5801*	TTCGGTTATCGATGAGGAGG	CATCATTGCGCCATGTACTC	92	(AGG)8	3	SSR	
136	RM571	GGAGGTGAAAGCGAATCATG	CCTGCTGCTCTTTCATCAGC	188	(GT)11(AG)13	3	SSR	

137	RM130	TGTTGCTTGCCCTCACGCGAAG	GGTCGCGTGCTTGGTTTGGTTC	85	(GA)10	3	SSR	
138	RM5686	CTCTTCTATGCATATTGCCA	ATAAACTGAGGGGCGATATA	149	(AAT)17	3	SSR	Polymorphic
139	R3M10*	CCGAGTACCATTGCTTTC	CTGCCATAGTTACTGCTCTGTT			3	InDel	
140	R3M23*	TGCTTACAAGGGTCCAAT	GGAGGTGCCTACCAAGAG			3	InDel	Polymorphic
141	R3M30*	AGGCTAAGTGAAGAAATAATAAG	CTCCGTATTCATTACTGGTTG			3	InDel	
142	R3M37*	GCATTGAATTGTACTCTTATTATAT	ACGAATCAAAAGGAGACTAAAAT			3	InDel	
143	R3M53*	ACACTGGCTACGGCAAAG	TTTGTTCGGGAATAATGATGC			3	InDel	
144	RM335	GTACACACCCACATCGAGAAG	GCTCTATGCGAGTATCCATGG	104	(CTT)25	4	SSR	
145	RM127*	GTGGGATAGCTGCGTCGCGTCG	AGGCCAGGGTGTTGGCATGCTG	223	(AGG)8	4	SSR	
146	RM348*	CCGCTACTAATAGCAGAGAG	GGAGCTTTGTTCTTGCGAAC	136	(CAG)7	4	SSR	
147	RM261	CTACTTCTCCCCTTGTGTCG	TGTACCATCGCCAAATCTCC	125	C9(CT)8	4	SSR	
148	RM8213	AGCCCAGTGATACAAAGATG	GCGAGGAGATACCAAGAAAG	177	(TC)10	4	SSR	
149	RM280*	ACACGATCCACTTTGCGC	TGTGTCTTGAGCAGCCAGG	155	(GA)16	4	SSR	
150	RM7563*	CCACCGCTCGTAGAAAAAAC	GGGTTGAGATGCCTGTGC	138	(TCCA)7	4	SSR	
151	RM17067*	CGCCTAACTCCTCGATCCAACG	TGAGGGTGAGGGTGGTGAGG	232	(GCG)9	4	SSR	
152	RM1100*	GAAAGAGCGAAGGCGGTG	TCTCTGTCTCTCTCGCTCTCG	130	(AG)12	4	SSR	
153	RM307*	GTACTACCGACCTACCGTTCAC	CTGCTATGCATGAACTGCTC	174	(AT)14(GT)21	4	SSR	
154	RM3648	TACCCTTTCTTCCCCAAACC	ACCTCCTCCTCCACTTCTCC	186	(GA)14	4	SSR	
155	RM17071	ATCTCTAGGGCCCTGATTACG	GTTGTTTGAGCTTGAGCTTACC	460	(TA)20	4	SSR	
156	RM16278*	AGTGATAAGCAAACGTACCCATCG	CCCTCGCCTGTAGCTATGAACG	153	(AT)16	4	SSR	
157	RM5611	ACGACCATAGAAGTTTGCCC	CTGCAGCTGCAACACCAC	162	(AAG)9	4	SSR	
158	RM303	GCATGGCCAAATATTAAAGG	GGTTGGAAATAGAAGTTCGGT	200	[AC(AT)2-10]9(GT)7(ATGT)6	4	SSR	Polymorphic
159	RM16956*	CCAAATCTATAGCTCTCCTTCTCC	ATACCAACTCTCCCAAGAACTCC	384	(CTT)7	4	SSR	
160	RM3742	CTCTTCATCCCCCAAGCC	GAGAAGAAGAACAGAGCTGCG	76	(GA)17	4	SSR	
161	RM518	CTCTTCACTCACTCACCATGG	ATCCATCTGGAGCAAGCAAC	171	(TC)15	4	SSR	
162	RM567	ATCAGGGAAATCCTGAAGGG	GGAAGGAGCAATCACCACTG	261	(GA)21	4	SSR	
163	RM5709	CTGAATTTATTATAGGACGGAAG	CATAGTATTGGATTGGACACG	163	(AAT)22	4	SSR	
164	RM349	TTGCCATTCGCGTGGAGGCG	GTCCATCATCCCTATGGTCG	136	(GA)16	4	SSR	
165	RM273	GAAGCCGTCGTGAAGTTACC	GTTTCCTACCTGATCGCGAC	207	(GA)11	4	SSR	Polymorphic
166	RM16686	GGCACTGCTTGCATATGGATCG	TGCCGGCGAACTTATCCTCTCC	93	(GGA)10	4	SSR	
167	RM124*	ATCGTCTGCGTTGCGGCTGCTG	CATGGATCACCGAGCTCCCCCC	271	(TC)10	4	SSR	
168	RM16672	CTGATCCAGCGGTGATGATGG	CTGCAGCTGTCCTCCCTTAGCC	69	(GGA)7	4	SSR	
169	RM5633	GTGTAGCTGCTAGGCCGAAC	TTCCTTTCGCTACGTTGGAC	211	(AAG)12	4	SSR	
170	R4M13*	TACACGGTAGACATCCAACA	ATGATTTAACCGTAGATTGG			4	InDel	
171	R4M17*	AGTGCTCGGTTTTGTTTTC	GTCAGATATAATTGATGGATGTA			4	InDel	

172	R4M30*	GCTTCTCCTGGTTGTATGC	AAAATAGGGAGGCAGATAGAC			4	InDel	
173	R4M43*	CTTGAACCTGAGTGAGTGG	CGATGAAAATGATGTCTA			4	InDel	
174	R4M50*	TTTTGTGAAACTTGACCCTC	GCGTCCATGTCTTTATTGTG			4	InDel	
175	RM440	CATGCAACAACGTCACCTTC	ATGGTTGGTAGGCACCAAAG	169	(CTT)22	5	SSR	
176	RM169*	TGGCTGGCTCCGTGGGTAGCTG	TCCCGTTGCCGTTCATCCCTCC	167	(GA)12	5	SSR	
177	RM122*	GAGTCGATGTAATGTCATCAGTGC	GAAGGAGGTATCGCTTTGTTGGAC	227	(GA)7A(GA)2A(GA)11	5	SSR	
178	RM334*	GTTCAGTGTTCAGTGCCACC	GACTTTGATCTTTGGTGGACG	182	(CTT)20	5	SSR	Polymorphic
179	RM163	ATCCATGTGCGCCTTTATGAGGA	CGCTACCTCCTTCACTTACTAGT	124	(GGAGA)4(GA)11C(GA)20	5	SSR	
180	RM164	TCTTGCCCGTCACTGCAGATATCC	GCAGCCCTAATGCTACAATTCTTC	246	(GT)16TT(GT)4	5	SSR	
181	RM305	TACTGCCAAAGGCGAGCTTC	GTGAGAGGCTACAGCTAACC	203	(GT)4+degener	5	SSR	
182	RM430	AAACAACGACGTCCCTGATC	GTGCCTCCGTGGTTATGAAC	173	(GA)25	5	SSR	
183	RM574*	GGCGAATTCTTTGCACTTGG	ACGGTTTGGTAGGGTGTCAC	155	(GA)11	5	SSR	
184	RM480*	GCTCAAGCATTCTGCAGTTG	GCGCTTCTGCTTATTGGAAG	225	(AC)30	5	SSR	Polymorphic
185	RM13	TCCAACATGGCAAGAGAGAG	GGTGGCATTCGATTCCAG	141	(GA)6-(GA)16	5	SSR	
186	RM7029*	GATTCCCTGCAGGAACAATG	CAAGGCAGACAACAACATGG	158	(AAAT)7	5	SSR	
187	RM7452	GAGGCCATGAACGGTCAC	ACCCAATTATGGTAGCGTGC	94	(TAAT)9	5	SSR	
188	RM26	GAGTCGACGAGCGGCAGA	CTGCGAGCGACGGTAACA	112	(GA)15	5	SSR	
189	RM178*	TCGCGTGAAAGATAAGCGGCGC	GATCACCGTTCCCTCCGCCTGC	117	(GA)5(AG)8	5	SSR	
190	RM507*	CTTAAGCTCCAGCCGAAATG	CTCACCCTCATCATCGCC	258	(AAGA)7	5	SSR	
191	RM413*	GGCGATTCTTGGATGAAGAG	TCCCCACCAATCTTGTCTTC	79	(AG)11	5	SSR	
192	RM249	GGCGTAAAGGTTTTGCATGT	ATGATGCCATGAAGGTCAGC	121	(AG)5A2(AG)14	5	SSR	
193	R5M13*	GAGAAAGAGTGGAAGGAG	AGTATCGTCAGGAGGGTC			5	InDel	Polymorphic
194	R5M30*	CTCAATTTCACCCATCCC	CGCTCCGTCTCCAACCTC			5	InDel	
195	RM19696	GCTTGGCATTTACTCTCCGTTC	GGAGATGATGGGACTGCAATAGG	535	(AT)42	6	SSR	
196	RM30*	GGTTAGGCATCGTCACGG	TCACCTCACCACACGACACG	105	(AG)9A(GA)12	6	SSR	
197	RM20757	ACAAGAATCCAGCACGTCACTCC	CTCTCCTGTTTGAACGGTTGTGG	526	(TA)10	6	SSR	
198	RM345	ATTGGTAGCTCAATGCAAGC	GTGCAACAACCCCACATG	167	(CTT)9	6	SSR	
199	RM204*	GTGACTGACTTGGTCATAGGG	GCTAGCCATGCTCTCGTACC	169	(CT)44	6	SSR	Polymorphic
200	RM19974	CTTGTCAAGTAAGCAACCGTATCC	CTCATTTGGCACAAACACATCC	593	(AT)14	6	SSR	
201	RM19629	CAATTTGGAAGTTGAGCCATCG	GAGTAGCTTGGCCCATAATTTGC	331	(TTA)30	6	SSR	
202	RM400	ACACCAGGCTACCCAAACTC	CGGAGAGATCTGACATGTGG	321	(ATA)63	6	SSR	
203	RM588	GTTGCTCTGCCTCACTCTTG	AACGAGCCAACGAAGCAG	126	(TGC)9	6	SSR	Polymorphic
204	RM586	ACCTCGCGTTATTAGGTACCC	GAGATACGCCAACGAGATACC	271	(CT)23	6	SSR	Polymorphic
205	RM587	ACGCGAACAAATTAACAGCC	CTTTGCTACCAGTAGATCCAGC	217	(CTT)18	6	SSR	Polymorphic
206	RM589	ATCATGGTCGGTGGCTTAAC	CAGGTTCCAACCAGACACTG	186	(GT)24	6	SSR	Polymorphic

207	RM340*	GGTAAATGGACAATCCTATGGC	GACAAATATAAGGGCAGTGTGC	163	(CTT)8T3(CTT)14	6	SSR	
208	RM103*	CTTCCAATTCAGGCCGGCTGGC	CGCCACAGCTGACCATGCATGC	336	(GAA)5	6	SSR	
209	RM217	ATCGCAGCAATGCCTCGT	GGGTGTGAACAAAGACAC	133	(CT)20	6	SSR	
210	RM170	TCGCGCTTCTTCCTCGTCGACG	CCCGCTTGCAGAGGAAGCAGCC	121	(CCT7	6	SSR	
211	RM276	CTCAACGTTGACACCTCGTG	TCCTCCATCGAGCAGTATCA	149	(AG)8A3(GA)33	6	SSR	
212	RM439*	TCATAACAGTCCACTCCCCC	TGGTACTCCATCATCCCATG	269	(AAT)13	6	SSR	Polymorphic
213	RM19406	AGGTTGGCAATGGAATCTTCAGC	ATTCCCATAGGCCACACAGATCC	383	(AT)21	6	SSR	
214	RM111	CACAACCTTTGAGCACCGGGTC	ACGCCTGCAGCTTGATCACCGG	124	(GA)9	6	SSR	
215	RM8250*	AACCTAAAGGGCAGTTTCC	GCGATAAGTTTCTTGTTGATG	171	(AC)13	6	SSR	Polymorphic
216	RM3827	GGACGGATTGTAGGTAGGAC	CCTTTCTTCAATCTGCATTC	160	(GA)21	6	SSR	
217	RM121	ACCGTCGCCTTCCACTTTCCCC	TTCGGGGTTGCCGGTGATGTTG	170	(CT)7	6	SSR	
218	RM133*	TTGGATTGTTTTGCTGGCTCGC	GGAACACGGGGTCGGAAGCGAC	230	(CT)8	6	SSR	
219	RM510*	AACCGGATTAGTTTCTCGCC	TGAGGACGACGAGCAGATTC	122	(GA)15	6	SSR	
220	RM162*	GCCAGCAAAACCAGGGATCCGG	CAAGGTCTTGTGCGGCTTGCGG	229	(AC)20	6	SSR	
221	RM225	TGCCCATATGGTCTGGATG	GAAAGTGGATCAGGAAGGC	140	(CT)18	6	SSR	Polymorphic
222	RM343	CCACGAACCCCTTTGCATC	GTGATGATGCGTCGGTTG	233	(CAT)5(CAC)5CAT(CAC)4	6	SSR	
223	RM6836*	TGTTGCATATGGTGCTATTTGA	GATACGGCTTCTAGGCCAAA	240	(TCT)14	6	SSR	
224	RM527	GGCTCGATCTAGAAAATCCG	TTGCACAGGTTGCGATAGAG	233	(GA)17	6	SSR	Polymorphic
225	RM115	TTGCCGCAGTGGCCGTTACCAC	AGGAGGCGGCGGAAATGGAAGG	190	(AG)7	6	SSR	
226	RM3431	ATCCAAATCCAATGGTGC	GCGAAAGGGAACATTCTG	161	(CT)18	6	SSR	Polymorphic
227	RM6836	TGTTGCATATGGTGCTATTTGA	GATACGGCTTCTAGGCCAAA	240	(TCT)14	6	SSR	
228	RM3805*	AGAGGAAGAAGCCAAGGAGG	CATCAACGTACCAACCATGG	110	(GA)19	6	SSR	Polymorphic
229	RM8225*	TGTTGCATATGGTGCTATTTGA	GATACGGCTTCTAGGCCAAA	240	(TCT)14	6	SSR	
230	RM5963*	CGAAAAGTGGGAAGCAAATG	GCGTACCCCTAGTGGCTGTA	196	(CAG)9	6	SSR	
231	RM19814	GGGTGAGGAAATGGGAGAGAGG	AAGCAACACACTGGAGAAGTGAGG	230	(GGT)7	6	SSR	
232	RM412	CACTTGAGAAAGTTAGTGCAGC	CCCAAACACACCCAAATAC	198	(GA)22	6	SSR	
233	R6M14*	AAATGTCCATGTGTTTGCTTC	CATGTGTGGAATGTGGTTG			6	InDel	
234	R6M44*	TTAGGAATAAAGGCTGGATA	TTACCGTTAATAGGTGGAA			6	InDel	
235	Si9337	AGATGTCCCTTCACTTCAGC	CGAAACGGCCCTTGATCC			6	InDel	
236	Si9575	GCGCACACGGAGAACACC	ACAGCTCACGTATAAATGTGAACGA			6	InDel	
237	Si9653	ACTGGATGTAACTATTGTATTGGCTA	GTCACACCGTCAGACCAT			6	InDel	
238	RM182	TGGGATGCAGAGTGCAGTTGGC	CGCAGGCACGGTGCCTTGTAAG	346	(AT)16	7	SSR	
239	RM21665	GTACTCCTACGATGCCAATGTGC	TGGCACTACTCCTACTCCACACC	133	(TC)11	7	SSR	
240	RM455*	AACAACCCACCACCTGTCTC	AGAAGGAAAAGGGCTCGATC	131	(TTCT)5	7	SSR	
241	RM11*	TCTCCTCTTCCCCCGATC	ATAGCGGGCGAGGCTTAG	140	(GA)17	7	SSR	

242	RM5344	ACGAACGGGAGCAAGGTC	CTCTCAACCAAGACGCCTTC	116	(TC)13	7	SSR	
243	RM7087	TAGCCTTGTTCCATCCATCC	CTTCTCCCTCTCCTCCTTCC	114	(AGAT)6	7	SSR	
244	RM1306	TGCCAATTACCTTCCCGTAC	TGCTCCGTATTGCTGCTATG	105	(AG)18	7	SSR	
245	RM125*	ATCAGCAGCCATGGCAGCGACC	AGGGGATCATGTGCCGAAGGCC	127	(GCT)8	7	SSR	
246	RM10	TTGTCAAGAGGAGGCATCG	CAGAATGGGAAATGGGTCC	159	(GA)15	7	SSR	
247	RM21653	TGTAAATTGTGCACGGTGATCC	GGAAGGTTGTGATTGCTAAAGAGG	246	(TA)13	7	SSR	
248	RM172	TGCAGCTGCGCCACAGCCATAG	CAACCACGACACCGCCGTGTTG	159	(AGG)6	7	SSR	
249	RM429	TCCCTCCAGCAATGTCTTTC	CCTTCATCTTGCTTTCCACC	159	(TG)10	7	SSR	
250	RM180	CTACATCGGCTTAGGTGTAGCAACACG	ACTTGCTCTACTTGTGGTGAGGGACTG	110	(ATT)10	7	SSR	
251	RM3743	TAGCCTTGTTCCATCCATCC	CTTCTCCCTCTCCTCCTTCC	180	(GA)17	7	SSR	
252	RM3691	GCTGATGGTCAAAGATCAGG	ATGTGTCTGCTGGCACAGAG	117	(GA)15	7	SSR	
253	RM5420	CCTGATCTCAACACACACGC	GAAGTCTTGTTGCGCGTATG	127	(TC)16	7	SSR	
254	RM1132	ATCACCTGAGAAACATCCGG	CTCCTCCCACGTCAAGGTC	93	(AG)12	7	SSR	
255	RM118*	CCAATCGGAGCCACCGGAGAGC	CACATCCTCCAGCGACGCCGAG	156	(GA)8	7	SSR	
256	RM248*	TCCTTGTGAAATCTGGTCCC	GTAGCCTAGCATGGTGCATG	102	(CT)25	7	SSR	
257	RM214*	CTGATGATAGAAACCTCTTCTC	AAGAACAGCTGACTTCACAA	112	(CT)14	7	SSR	Polymorphic
258	RM336	CTTACAGAGAAACGGCATCG	GCTGGTTTGTTTCAGGTTCG	154	(CTT)18	7	SSR	
259	RM10	TTGTCAAGAGGAGGCATCG	CAGAATGGGAAATGGGTCC	159	(GA)15	7	SSR	
260	RM3670	GATCAAGAAGCAGGACACCG	GATCTGGAGTCGCGTCAAAC	111	(GA)14	7	SSR	
261	RM5436*	CAAAGGGGGTGTCCTCTATG	GTTGCTCGTCCTACATGTGC	161	(TC)17	7	SSR	
262	RM2256*	GTGCTTGCATATAACCTATA	AGATCAACCTTCTTATTCAG	167	(AT)24	7	SSR	Polymorphic
263	RM5720*	CCTGATAAATTGACAGTTAC	GAGAGTAGGAGTTGATAACA	196	(AAT)22	7	SSR	
264	RM1362	TGATCTAAACAGGCCCTTAG	CATCATCAAGACCACACATC	227	(AG)25	7	SSR	Polymorphic
265	RM1253	CTGAACTTGCCTGAGAACTC	GACGACCTCTCCATGCTCG	175	(AG)16	7	SSR	
266	R7M7*	ACCTTCCCTCCCCTTTTGAT	AACTTGGTCTTCCTGTTTTATTG			7	InDel	
267	R7M20*	GTTTTGTGCATTCCTTTAC	TTTATGACATTTTGACCG			7	InDel	
268	R7M37*	CAGCCCTAAATCTAAATACCC	ACGTTGAGACAGGCGAGC			7	InDel	
269	RM447*	CCCTTGTGCTGTCTCCTCTC	ACGGGCTTCTTCTCCTTCTC	111	(CTT)8	8	SSR	
270	RM284*	ATCTCTGATACTCCATCCATCC	CCTGTACGTTGATCCGAAGC	141	(GA)8	8	SSR	Polymorphic
271	RM72	CCGGCGATAAAACAATGAG	GCATCGGTCCTAACTAAGGG	166	(TAT)5C(ATT)15	8	SSR	
272	RM1235	AGCAGAGGAGGAGATGATGG	GGACCAAAACGAAGCTATCC	118	(AG)15	8	SSR	
273	RM149	GCTGACCAACGAACCTAGGCCG	GTTGGAAGCCTTTCCTCGTAACACG	253	(AT)10	8	SSR	
274	RM44*	ACGGGCAATCCGAACAACC	TCGGGAAAACCTACCCTACC	99	(GA)16	8	SSR	
275	RM210	TCACATTCGGTGGCATTG	CGAGGATGGTTGTTCACTTG	140	(CT)23	8	SSR	
276	RM556	ACTCCAAACCTCACTGCACC	TAGCACACTGAACAGCTGGC	93	(CCAG)6	8	SSR	

277	RM5499	TGGAGTACGACGTGATCGTG	CAGAAACGGGAGGGGATC	240	(TC)25	8	SSR	Polymorphic
278	RM408*	CAACGAGCTAACTTCCGTCC	ACTGCTACTTGGGTAGCTGACC	128	(CT)13	8	SSR	
279	RM152*	GAAACCACCACACCTCACCG	CCGTAGACCTTCTTGAAGTAG	151	(GGC)10	8	SSR	
280	RM531	GAAACATCCCATGTTCCCAC	TCGGTTTTTCAGACTCGGTC	128	(AT)15	8	SSR	
281	RM337	GTAGGAAAGGAAGGGCAGAG	CGATAGATAGCTAGATGTGGCC	192	(CTT)4-19-(CTT)8	8	SSR	
282	RM339	GTAATCGATGCTGTGGGAAG	GAGTCATGTGATAGCCGATATG	148	(CTT)8CCT(CTT)5	8	SSR	
283	RM25*	GGAAAGAATGATCTTTTCATGG	CTACCATCAAAACCAATGTTC	146	(GA)18	8	SSR	
284	RM159	GGGGCACTGGCAAGGGTGAAGG	GCTTGTGCTTCTCTCTCTCTCTCTCTCTC	248	(GA)19	8	SSR	
285	RM256	GACAGGGAGTGATTGAAGGC	GTTGATTTCGCCAAGGGC	127	(CT)21	8	SSR	
286	RM433*	TGCGCTGAACTAAACACAGC	AGACAAACCTGGCCATTCAC	224	(AG)13	8	SSR	
287	RM310	CCAAAACATTTAAAATATCATG	GCTTGTTGGTCATTACCATTC	105	(GT)19	8	SSR	
288	RM223	GAGTGAGCTTGGGCTGAAAC	GAAGGCAAGTCTTGGCACTG	165	(CT)25	8	SSR	
289	RM281	ACCAAGCATCCAGTGACCAG	GTTCTTCATACAGTCCACATG	138	(GA)21	8	SSR	
290	RM23478	CGACGCAGGGTTTAGATAGAGTGC	GTTCTCGTTCCGATGGCTAGACG	177	(GAG)7	8	SSR	
291	RM80	TTGAAGGCGCTGAAGGAG	CATCAACCTCGTCTTCACCG	142	(TCT)25	8	SSR	
292	GBR8001	CCATAGAGGCCTACAAGTAT	CCAGATGATAGAAGAGGTGT	160		8	SSR	Polymorphic
293	RM344	CAGAGACAATAGTCCCTGCAC	GTAGGAGGAGATGGATGATGG	163	(TTC)2-5-(CTT)3-(CTT)4	8	SSR	
294	RM515	GCTGACCAACGAACCTAGGCCG	GTTGGAAGCCTTTCCTCGTAACACG	211	(GA)11	8	SSR	Polymorphic
295	RM42	ATCCTACCGCTGACCATGAG	TTTGGTCTACGTGGCGTACA	166	(AG)6-(AG)2T(GA)5	8	SSR	
296	RM342	CCATCCTCCTACTTCAATGAAG	ACTATGCAGTGGTGTCACCC	141	(CAT)12	8	SSR	
297	RM38	ACGAGCTCTCGATCAGCCTA	TCGGTCTCCATGTCCCAC	250	(GA)16	8	SSR	Polymorphic
298	RM502	GCGATCGATGGCTACGAC	ACAACCCAACAAGAAGGACG	266	(TG)10	8	SSR	
299	RM547	TAGGTTGGCAGACCTTTTCG	GTCAAGATCATCCTCGTAGCG	235	(ATT)19	8	SSR	Polymorphic
300	RM230*	GCCAGACCGTGGATGTTC	CACCGCAGTCACTTTTCAAG	257	(AGG)4(GA)9A(AG)13	8	SSR	
301	RM5556	ATCTCCCTCCCTCTCCTCAC	TCCACACCTTCACAGTTGAC	102	(TG)15	8	SSR	
302	RM5432	GTTTCCCCACTTATCTCCCC	AAGCGAGGAGGGGTTTAGAG	216	(TC)16	8	SSR	
303	RM3791	GTAGAGGCCTACAAGTATCCTCG	ACCCACATAATTCACAGGGG	239	(AG)20	8	SSR	Polymorphic
304	RM210	TCACATTCGGTGGCATTG	CGAGGATGGTTGTTCACTTG	140	(CT)23	8	SSR	
305	RM3459	ATGGACTTTCGAGAATGTTG	GAGTACGAAATGAAGGCAAG	186	(CT)20	8	SSR	
306	Aro7	ATTTGCCTCCTGAGTCTG	GAGGATGGGGAAGATAAA	302	(AGG)9	8	SSR	
307	RM23120	AACTGTTGGATCGACAAGACCTTCC	ACGGCGTTAAGCTAGACAGACAGAGC	435	(TA)28	8	SSR	
308	R8M23*	CCTATTCACTCTACCGACAT	GTTTAGTTCCCATTGCTTT			8	InDel	
309	R8M33*	CGAAAGAGGAGAGGGGTAGT	CGAAAACGAGAAACAAATA			8	InDel	
310	RM296	CACATGGCACCAACCTCC	GCCAAGTCATTCACTACTCTGG	123	(GA)10	9	SSR	
311	RM205	CTGGTTCTGTATGGGAGCAG	CTGGCCCTTCACGTTTCAGTG	122	(CT)25	9	SSR	

312	RM105*	GTCGTCGACCCATCGGAGCCAC	TGGTCGAGGTGGGGATCGGGTC	134	(CCT)6	9	SSR	
313	RM434	GCCTCATCCCTCTAACCCTC	CAAGAAAGATCAGTGCGTGG	152	(TC)12	9	SSR	
314	RM257	CAGTTCCGAGCAAGAGTACTC	GGATCGGACGTGGCATATG	147	(CT)24	9	SSR	
315	RM24181	ATGCCAGCAAGAAGGCAAGAACG	GACAAGAGTCGCCGAGGCATCC	107	(CT)17	9	SSR	
316	RM24334	GAACGGTTTGAGGAAGAAGAAGACG	ATCCATCCACGACACACCATCC	197	(CT)12	9	SSR	
317	RM242	GGCCAACGTGTGTATGTCTC	TATATGCCAAGACGGATGGG	225	(CT)26	9	SSR	
318	RM524	TGAAGAGCAGGAACCGTAGG	TCTGATATCGGTTCCTTCGG	198	(AT)11	9	SSR	
319	RM566	ACCCAACTACGATCAGCTCG	CTCCAGGAACACGCTCTTTC	239	(AG)15	9	SSR	
320	RM24390	GAAGCTCTGGATTTGCGACATGG	GAGGGCGTGAGGTGAAGTTGG	90	(CT)11	9	SSR	
321	RM1896	GGACAGGGTAAAGTGTTAGA	CCTAAGACCTATCAACTCCA	108	(AT)18	9	SSR	
322	RM215*	CAAAATGGAGCAGCAAGAGC	TGAGCACCTCCTTCTCTGTAG	148	(CT)16	9	SSR	
323	RM316*	CTAGTTGGGCATACGATGGC	ACGCTTATATGTTACGTCAAC	192	(GT)8-(TG)9(TTTG)4(TG)4	9	SSR	Polymorphic
324	RM160	AGCTAGCAGCTATAGCTTAGCTGGAGATCG	TCTCATCGCCATGCGAGGCCTC	131	(GAA)23	9	SSR	
325	RM3600	TGCCCACACATGATGAGC	AACGGGCAAGAGATCTTCTG	92	(GA)13	9	SSR	
326	RM24325	CGTCGTACTTGGTGTGTTGATCC	AGCCAACCAAACCAACCAACC	181	(CT)10	9	SSR	
327	RM444	GCTCCACCTGCTTAAGCATC	TGAAGACCATGTTCTGCAGG	162	(AT)12	9	SSR	Polymorphic
328	RM410*	GCTCAACGTTTCGTTCCTG	GAAGATGCGTAAAGTGAACGG	183	(TA)13	9	SSR	
329	RM201*	CTCGTTTATTACCTACAGTACC	CTACCTCCTTTCTAGACCGATA	158	(CT)17	9	SSR	
330	RM321	CCAACACTGCCACTCTGTTC	GAGGATGGACACCTTGATCG	200	(CAT)5	9	SSR	
331	RM8300	GCT AGT GCA GGG TTG ACA CA	CTC TGG CCG TTT CAT GGT AT	180	(ACCATT)n	9	SSR	Polymorphic
332	Sub AB1	CATGTTCCATAGCCATCGACT	GAGCGAAGAGAGCTACCTGAA			9	InDel	
333	ART5	CAGGGAAAGAGATGGTGGA	TTGGCCCTAGGTTGTTTCAG			9	InDel	
334	R9M10*	CTTTGGATTCAGGGGGA	AACTTGAAACGGAGGCAG			9	InDel	
335	R9M20*	ACTGCTTTGATGGCTTGTG	CTCCCCAAACTGAATCC			9	InDel	
336	R9M30*	CTCACCTACCTAAAACCCAAC	CCACCCAAATCTGATACTG			9	InDel	
337	R9M42*	CTATAAGACCAAAACGAAAACT	GAAAACCATTGTGTCACTGTA			9	InDel	
338	RM222	CTTAAATGGGCCACATGCG	CAAAGCTTCCGGCCAAAAG	213	(CT)18	10	SSR	
339	RM311	TGGTAGTATAGGTACTAAACAT	TCCTATACACATACAAACATAC	179	(GT)3(GTAT)8(GT)5	10	SSR	
340	RM496	GACATGCGAACAACGACATC	GCTGCGGCGCTGTTATAC	267	(TC)14	10	SSR	
341	RM271*	TCAGATCTACAATTCCATCC	TCGGTGAGACCTAGAGAGCC	101	(GA)15	10	SSR	
342	RM25539	AAGCCCTATCATTTGTCCTTCC	CACCCATCTTGCACTTATCAGC	282	(AT)35	10	SSR	
343	RM304	TCAAACCGGCACATATAAGAC	GATAGGGAGCTGAAGGAGATG	160	(GT)2(AT)10(GT)33	10	SSR	
344	RM269	GAAAGCGATCGAACCAGC	GCAAATGCGCCTCGTGTC	182	(GA)17	10	SSR	
345	RM171*	AACGCGAGGACACGTACTTAC	ACGAGATACGTACGCCTTTG	328	(GATG)5	10	SSR	
346	RM184	ATCCCATTCGCCAAAACCGGCC	TGACACTTGGAGAGCGGTGTGG	219	(CA)7	10	SSR	

347	RM333	GTACGACTACGAGTGTCACCAA	GTCTTCGCGATCACTCGC	191	(TAT)19(CTT)19	10	SSR	Polymorphic
348	RM239	TACAAAATGCTGGGTACCCC	ACATATGGGACCCACCTGTC	144	(AG)5TG(AG)2	10	SSR	
349	RM258	TGCTGTATGTAGCTCGCACC	TGGCCTTTAAAGCTGTCGC	148	(AG)5TG(AG)2	10	SSR	
350	RM467*	GGTCTCTCTCTCTCTCTCTCTCTC	CTCCTGACAATTCAACTGCG	221	(TC)21	10	SSR	Polymorphic
351	RM228*	CTGGCCATTAGTCCTTGG	GCTTGCGGCTCTGCTTAC	154	(CA)6(GA)36	10	SSR	
352	RM7020	GTAACGCTGCTTGAATTGCC	GTCCATAATCCCCTCTTGAC	195	(AAAG)7	10	SSR	Polymorphic
353	RM596	ATCTACACGGACGAATTGCC	AGAAGCTTCAGCCTCTGCAG	188	(GAC)10	10	SSR	
354	RM5304	CAGCCCATCTCTCTCCTCTG	GATAGCAGGAAGAGGCGTTG	143	(TC)12	10	SSR	
355	RM474*	AAGATGTACGGGTGGCATTC	TATGAGCTGGTGAGCAATGG	252	(AT)13	10	SSR	
356	RM484*	TCTCCCTCCTCACCATTGTC	TGCTGCCCTCTCTCTCTCTC	299	(AT)9	10	SSR	
357	R10M10*	GAATACAACCCCCTAAAAAC	ATGGACCGTTGAGGAGAC			10	InDel	
358	R10M17*	TGAACAATAAACCACAGAAGCA	CCCTTTATTCCCTCCTTTG			10	InDel	Polymorphic
359	R10M30*	CCCTAAAAATAGAGCAACCT	ACCCATAATACTACCAATCAAC			10	InDel	
360	R10M40*	GTCCCTAGGCCATCTCTTG	GCGAATAGGGGTGGACAG			10	InDel	
361	RM286	GGCTTCATCTTTGGCGAC	CCGGATTCACGAGATAAACTC	110	(GA)16	11	SSR	Polymorphic
362	RM6327	CAGCCTAGGGCGTCATAGAC	GATTGGGTGATGGATAGCAC	212	(CTT)18	11	SSR	
363	RM224	ATCGATCGATCTTCACGAGG	TGCTATAAAAGGCATTCGGG	157	(AAG)8(AG)13	11	SSR	
364	RM209	ATATGAGTTGCTGTCGTGCG	CAACTTGCATCCTCCCCTCC	134	(CT)18	11	SSR	
365	RM167	GATCCAGCGTGAGGAACACGT	AGTCCGACCACAAGGTGCGTTGTC	128	(GA)16	11	SSR	
366	RM206	CCCATGCGTTTAACTATTCT	CGTTCCATCGATCCGTATGG	147	(CT)21	11	SSR	
367	RM202	CAGATTGGAGATGAAGTCCTCC	CCAGCAAGCATGTCAATGTA	189	(CT)30	11	SSR	
368	RM21	ACAGTATTCCGTAGGCACGG	GCTCCATGAGGGTGGTAGAG	157	(GA)18	11	SSR	
369	RM6094	TGCTTGATCTGTGTTCGTCC	TAGCAGCACCAGCATGAAAG	182	(CCT)13	11	SSR	Polymorphic
370	RM552*	CGCAGTTGTGGATTTCAGTG	TGCTCAACGTTTGACTGTCC	195	(TAT)13	11	SSR	
371	RM536*	TCTCTCCTCTTGTTTGGCTC	ACACACCAACACGACCACAC	243	(CT)16	11	SSR	
372	RM287*	TTCCCTGTTAAGAGAGAAATC	GTGTATTTGGTGAAAGCAAC	118	(GA)21	11	SSR	
373	RM144*	TGCCCTGGCGCAAATTTGATCC	GCTAGAGGAGATCAGATGGTAGTGCATG	237	(ATT)11	11	SSR	
374	RM229	CACTCACACGAACGACTGAC	CGCAGGTTCTTGTGAAATGT	116	(TC)11(CT)5C3(CT)5	11	SSR	
375	RM332*	GCGAAGGCGAAGGTGAAG	CATGAGTGATCTCACTCACCC	183	(CTT)5-12-(CTT)14	11	SSR	
376	RM254*	AGCCCCGAATAAATCCACCT	CTGGAGGAGCATTTGGTAGC	165	(TC)6ATT(CT)11	11	SSR	
377	R11M23*	AAGGTTGACAAGGACAGAAG	TCGCAGGAATGGATAAAA			11	InDel	Polymorphic
378	R11M40*	AAGAAAAATATCTATTGAGGAGTG	GGAGGACCATAAATGACGG			11	InDel	
379	RM28199	CGGCTTAGGGAGCGTCTGTAGG	GCATGCTAGTATGGCCACCATATTCC	179	(ATAG)5	12	SSR	
380	RM313	TGCTACAAGTGTTCTTCAGGAC	GCTCACCTTTTGTGTTCCAC	111	(GT)6CA(CG)5-6-(GT)8	12	SSR	
381	RM247	TAGTGCCGATCGATGTAACG	CATATGGTTTTGACAAAGCG	131	(CT)16	12	SSR	Polymorphic

382	RM28130	CAGCAGACGTTCCGGTTCTACTCG	AGGACGGTGGTGGTGATCTGG	175	(GAG)7	12	SSR	
383	RM6296	TCTTGCCTCGCTAGGGTTAG	CCCACGTTTCTCTTGTCCTC	155	(TCT)8	12	SSR	
384	RM519*	TGCCCTGTTATTTTCTTCTCTC	GGTGATCCTTTCCCATTTCA	122	(AAG)8	12	SSR	
385	RM28166	TGCTTGCAAACATTGCTTCTGG	ACTGATGTACTGAACACGGGAAGG	194	(CT)12	12	SSR	
386	RM235	AGAAGCTAGGGCTAACGAAC	TCACCTGGTCAGCCTCTTTC	124	(CT)24	12	SSR	
387	RM20*	ATCTTGTCCCTGCAGGTCAT	GAAACAGAGGCACATTTCATTG	140	(ATT)14	12	SSR	Polymorphic
388	RM28099	TGTGCGGATGCGGGTAAGTCC	CCACCTGTCAACCACCGAAACC	120	(GCG)7	12	SSR	
389	RM1103	CAGCTGCTGCTACTACACCG	CTACTCCACGTCCATGCATG	216	(AG)12	12	SSR	
390	RM260*	ACTCCACTATGACCCAGAG	GAACAATCCCTTCTACGATCG	111	(CT)34	12	SSR	Polymorphic
391	RM270*	GGCCGTTGGTTCTAAAATC	TGCGCAGTATCATCGGCGAG	108	(GA)13	12	SSR	
392	RM1261	GTCCATGCCCAAGACACAAC	GTTACATCATGGGTGACCCC	167	(AG)16	12	SSR	
393	RM28076	GGGACTTGGGACCAGTTTATGG	TCAGGTCTGTTGGATTCCATGC	289	(CT)13	12	SSR	
394	RM28048	TTCAGCCGATCCATTCAATTCC	GCTATTGGCCGGAAAGTAGTTAGC	93	(CGC)8	12	SSR	
395	RM415	CTTCGATCCATCATCCATGG	ATTGCTGTACGCAGTTTCGG	227	(AT)21	12	SSR	
396	RM12	TGCCCTGTTATTTTCTTCTCTC	GGTGATCCTTTCCCATTTCA	184	(GA)21	12	SSR	
397	RM19*	CAAAAACAGAGCAGATGAC	CTCAAGATGGACGCCAAGA	226	(ATC)10	12	SSR	
398	RM511	CTTCGATCCGGTGACGAC	AACGAAAGCGAAGCTGTCTC	130	(GAC)7	12	SSR	
399	R12M10*	ATCATTTCAGCCTGTGCC	AGCTTAATAGGGGGGACG			12	InDel	
400	R12M27*	ATTTCATTGCCATCAGTT	GTAATCTTCTATCCGTTCA			12	InDel	Polymorphic
401	R12M33*	TTGATGATAGTATTTGCTGATG	AGATAGTGTCGGCGGTGG			12	InDel	
402	R12M43*	CCGCCGAGAAGAAACAAAG	CCCAAGAACAGGATTACA			12	InDel	Polymorphic
*150 markers used for detection of genomic integrity of PPIS mutant with its parent, Kon Joha								

Supplementary Table S2 Rice chromosome 6-based SSR markers.

Sl. No.	Marker Name	Forward Primer Sequence (5'-3')	Reverse Primer Sequence (5'-3')	Repeat motif	Position start	Position end	Product size	Polymorphism status
1	RM19545	TAGTTGGGTGGAAATGGTCATCC	TAAGACGAGTGGTCACACAGTGC	(TAA)21	4999931	5000396	465	Non-polymorphic
2	RM19549	CCTGGTACTAACCATGTGATTGAGC	AACGTCAGAGTCTCACCACAAGC	(AT)35	5046744	5046995	251	Non-polymorphic
3	RM19555	GGCACTATATGTTGATCGCTAGTGG	ATGGTCCTTTGCAGGGATTACG	(AT)31	5233564	5233859	295	Non-polymorphic
4	RM19569	TATAAGGGTTGGGAACTTGTCC	CAAGCCCATTATGAGTTAGAGG	(AT)48	5390610	5390955	345	Non-polymorphic
5	RM3794	GCTGCTACTACCTCTGCCTCTTCC	TCAATCGATCCATCCATCTCTCC	(GA)19	5501421	5501564	144	Polymorphic
6	RM19586	ATGGCCCACAAGTCATTCTAAGG	CATGTCATGGAGACGAGGAAAGG	(TA)19	5558448	5558814	366	Non-polymorphic
7	RM19592	GTTGGCATCATCGCTCTTCTAGC	TAATTGCGTGCATCCATCTTCG	(TA)42	5667016	5667450	434	Polymorphic
8	RM2615	ATCTCGTTCATACTGCTTGACC	GACTGGTTTCCTTCATGTTACC	(AT)30	5958803	5959315	164	Non-polymorphic
9	RM19617	GAGGACGAAGGCGAGGAAGG	ATCACAGAGCGCTCGAGACAAGG	(CT)28	6151883	6152313	430	Non-polymorphic
10	RM19626	ATGGACAAATGCCCATACATGC	CCTATCTACGCGGAAAGTAAGGTACG	(TA)35	6287486	6287733	247	Non-polymorphic
11	RM19629	CAATTTGGAAGTTGAGCCATCG	GAGTAGCTTGGCCCATAATTTGC	(TTA)30	6299080	6299411	331	Non-polymorphic
12	RM19646	AGTAGAGACTAGGGTGCAAATACTGC	CTAGCTGACCCATGTTCTTCTAGC	(AG)27	6461642	6462188	546	Non-polymorphic
13	RM19666	CGTCAGCATCCTCTCTCGCTTTCC	CTGCGTGCTGCTCTTCCATCC	(TC)19	6948801	6949068	267	Non-polymorphic
14	RM19676	CCGTGTCAAGTAGTATTCTCACC	CCCTTAATACATATGCCAGTCC	(TA)33	7145797	7146240	443	Non-polymorphic
15	RM5855	CTCGTCCGTCTTTCTGCCTTACC	TGCTTCTCGCTTCGTGTAGAAGG	(ATA)29	7410636	7411193	229	Non-polymorphic
16	RM19696	GCTTGGCATTTACTCTCCGTTCC	GGAGATGATGGGACTGCAATAGG	(AT)42	7504223	7504758	535	Non-polymorphic
17	RM19713	CTTATCAGCCACACATTTGGAACC	CATATGGGCTCAGGGATTATTGG	(TAT)19	7812840	7813135	295	Non-polymorphic
18	RM19707	GCACTCGCATTAAATGTGCATACG	CTCCTTGTCCAGATGGAGGAACC	(TA)19	7687553	7688052	499	Non-polymorphic
19	RM19725	GATCCGAAGATGTGCCAGACAGG	GCCATGGCGAGGAGATTTGG	(TC)22	8122066	8122320	254	Polymorphic
20	RM19728	GGTCATGCATGGATGGAATATGG	GGTTGTAGGGCAGGACGGATAGG	(AT)29	8166877	8167336	459	Non-polymorphic
21	RM19744	CTAGACCAGATTGTGGATGAACG	GAAGTGGAAGAGATCCGCTAGG	(TA)30	8429752	8429943	191	Non-polymorphic
22	RM19758	GTTCTGATCGAGTGTGGTTTCG	ATGTGAGTCTTTGGATGCTTCG	(TA)28	8739516	8739900	384	Non-polymorphic
23	RM2523	TTTGTGGGCTAGGATTACCTCACG	CACGAATCCTATGACTGATCCAACG	(AT)28	8918950	8919095	146	Non-polymorphic
24	RM19771	AACCAATGCACACTTCTTCTGTGC	CAACTGTAGAGGTTGGAATGATCTGC	(AT)47	9032616	9032897	281	Non-polymorphic
25	RM19793	GTGGTAGTGGGCCTTGTCAGTTTGG	GGATCGGGTGGGAGTTTGTTGG	(TA)29	9559488	9559872	384	Non-polymorphic
26	RM19799	GAGAGACATGTTGTTGTGTTCTCC	CTGGTTGATGTTACCAATCACG	(TA)45	9676516	9676865	349	Non-polymorphic
27	RM527	CGGTTTGTACGTAAGTAGCATCAGG	TCCAATGCCAACAGCTATACTCG	(GA)17	9862291	9862523	233	Non-polymorphic

28	RM19819	CAAGGGATACATTGGGTTGTCG	TCCTCACAAATGGGAACTTAGGC	(AT)42	10477623	10477951	328	Non-polymorphic
29	RM19835	CGCTAGTGAAGCAATTCCTATGG	GATAGGGTGGGATACAACATAGACC	(TA)30	10822809	10823019	210	Non-polymorphic
30	RM5850	TTATACACAGATGACGCACACG	TGGGTTAAGGGACACACTTAGG	(ATA)27	10997667	10997866	181	Polymorphic
31	RM19849	GTTTAGTAGTTCGGTGATGTGC	GACTCCGAGTAAAGTTCATGG	(AT)34	11181474	11181968	494	Non-polymorphic
32	RM19866	GCTGAAACATGCCTGGTGATGG	CGCCTCCAACCACCCTATCG	(TA)29	11510644	11510922	278	Non-polymorphic
33	RM19902	ACGAGTGCAGACACAATCACATGC	CACATCAAAGACTAATGCGGAAGACC	(AT)43	12088484	12088715	231	Polymorphic
34	RM19912	CAACCTTTCCAAGGTCGAGTACG	AATGGTGGTACTGCCTTGTTTCC	(TTA)23	12274780	12274962	182	Non-polymorphic
35	RM19918	GTGCAATCCTGCTGTTGGAACC	CCAGCATTACATAGGGTTACCATGC	(AT)29	12320121	12320524	403	Non-polymorphic
36	RM19930	CTATCGGATGATCCACTGTCAGG	TAGAGGCCAGGGATGATGTCG	(AT)26	12620968	12621130	162	Non-polymorphic
37	RM19954	GGATTGACATTATTGCGGTGATCC	GCATTGTATGGTCGCACTGTGG	(AT)38	13032067	13032536	469	Non-polymorphic
38	RM19961	AATTCTTAGGGTCCGGATTACCG	GTAAACATGGGAAGTTGGGAACC	(AG)22	13194979	13195420	441	Non-polymorphic

Supplementary Table S3 Different allele-specific primer combination generated through WEBSNAPPER tool for Hd1 mutation detection.

Sl. No.	Primer name	Sequence (5' to 3')	Synthesis status	Polymorphism status
1	Wild1_FP	AGGGAGGCCAGGGTGCTCATG	Synthesized	Working
2	Wild2_FP	CAGGGAGGCCAGGGTGCTAAGG	Not synthesized	
3	Wild3_FP	CAGGGAGGCCAGGGTGCTCAAG	Not synthesized	
4	Wild4_FP	CAGGGAGGCCAGGGTGCTTAGG	Not synthesized	
5	Wild5_FP	GGGAGGCCAGGGTGCTCTGG	Not synthesized	
6	Mut1_FP	GGGAGGCCAGGGTGCTCGGT	Not synthesized	
7	Mut2_FP	CAGGGAGGCCAGGGTGCTCAAT	Synthesized	Not clearly polymorphic
8	Mut3_FP	GGGAGGCCAGGGTGCTCCGT	Synthesized	Working
9	Mut4_FP	CAGGGAGGCCAGGGTGCTCTGT	Not synthesized	
10	Mut5_FP	GACAGGGAGGCCAGGGTGCTAAGT	Not synthesized	
11	Mut6_FP	CAGGGAGGCCAGGGTGCTCATT	Not synthesized	
12	Common_RP	CTACTGTCAGATAGAGCTGCAGTGGAGAACATC	Synthesized	Working

Supplementary Table S4 DUS characteristics of the PPIS mutant and its parent *Kon Joha*.

Sl. No.	Characteristics	*Kon Joha*	PPIS mutant
**DUS characteristics (PPV & FRA, 2007)**			
1	Coleoptile colour	Green	Green
2	Basal leaf sheath colour	Green	Green
3	Leaf: Intensity of green colour	Medium	Medium
4	Leaf: Anthocyanin colouration	Absent	Absent
5	Leaf sheath: Anthocyanin colouration	Absent	Absent
6	Leaf: Pubescence of blade surface	Strong	Strong
7	Leaf: Auricles	Present	Present
8	Leaf: Anthocyanin colouration of auricles	Colourless	Colourless
9	Leaf: Collar	Present	Present
10	Leaf: Anthocyanin colouration of collar	Absent	Absent
11	Leaf: Ligule	Present	Present
12	Leaf: Shape of ligule	Split	Split
13	Leaf: Colour of ligule	White	White
14	Leaf: Length of blade	Long	Long
15	Leaf: Width of blade	Narrow	Narrow
16	Culm: Attitude	Semi erect	Semi erect
17	Time of heading (50% of plants with panicles)	Late	Medium
18	Flag leaf: Attitude of blade (early observation)	Semi erect	Semi erect
19	Spikelet: Density of pubescence of lemma	Medium	Medium
20	Male sterility	Absent	Absent
21	Lemma: Anthocyanin colouration of keel	Absent	Absent
22	Lemma: Anthocyanin colouration of area below apex	Absent	Absent
23	Lemma: Anthocyanin colouration of apex	Absent	Absent
24	Spikelet: Colour of stigma	White	White
25	Stem: Thickness	Thin	Thin
26	Stem: Length	Short	Short
27	Stem: Anthocyanin colouration of nodes	Absent	Absent
28	Stem: Anthocyanin colouration of internodes	Absent	Absent
29	Panicle: Length of main axis	Medium	Medium

30	Panicle: Curvature of main axis	Deflexed	Deflexed
31	Panicle: Number per plant	Medium	Medium
32	Spikelet: Colour of tip of lemma	White	White
33	Lemma and palea: Colour	Straw	Straw
34	Panicle: Awns	Absent	Absent
35	Panicle: Presence of secondary branches	Present	Present
36	Panicle: Secondary branching	Strong	Strong
37	Panicle: Attitude of branches	Semi-erect	Semi-erect
38	Panicle: Exertion	Well exerted	Well exerted
39	Time of maturity	Late	Medium
40	Leaf: Senescence	Medium	Medium
41	Sterile lemma: Colour	Straw	Straw
42	Grain weight of 1000 fully developed grains	Very low	Very low
43	Grain length (mm)	Short	Short
44	Grain width (mm)	Narrow	Narrow
45	Grain: Phenol reaction of lemma	Present	Present
46	Decorticated grain length	Short	Short
47	Decorticated grain width	Narrow	Narrow
48	Decorticated grain shape	Short bold	Short bold
49	Decorticated grain colour	White	White
50	Decorticated grain aroma	Present	Present
**DUS characteristics (Bioversity International, IRRI and WARDA, 2007)**			
51	Flag leaf: Attitude of blade (Late observation)	Descending	Descending
52	Leaf blade attitude	Erect	Erect
53	Lemma shape of the apiculus	Pointed	Pointed
54	Panicle threshability	Medium	Medium

Supplementary Table S5 The mean values for yield and yield component traits of the PPIS mutant and its parent *Kon Joha*.

Sl. No.	Trait	*Kon Joha*	PPIS mutant (JKOJM-250-22-17-125)
1	Days to 50% flowering	110.60 ± 0.60	100.70 ± 0.30 **
2	Days to maturity	146.50 ± 0.45	136.40 ± 0.43**
3	Plant height (cm)	136.69 ± 2.51	124.85 ± 1.46**
4	Productive tillers Plant^{-1}	13.10 ± 1.07	12.88 ± 0.71
5	Panicle length (cm)	28.40 ± 0.54	25.32 ± 0.50**
6	Panicle weight (g)	3.43 ± 0.11	3.33 ± 0.09
7	Yield Plant^{-1} (g)	12.64 ± 0.18	12.58 ± 0.40
8	Filled grains Panicle^{-1}	220.10 ± 13.25	190.50 ± 10.23*
9	Spikelet fertility %	90.70 ± 0.18	88.47 ± 0.24
10	1000-grain weights (g)	11.67 ± 0.09	11.58 ± 0.13
11	Grain length (mm)	6.23 ± 0.19	6.27 ± 0.17
12	Grain width (mm)	2.12 ± 0.05	2.13 ± 0.05

Note: *, ** Significantly different from *Kon Joha* at $P \leq 0.05$ and $P \leq 0.01$, respectively
